# Accurate Computation of Survival Statistics in Genome-Wide Studies

**DOI:** 10.1371/journal.pcbi.1004071

**Published:** 2015-05-07

**Authors:** Fabio Vandin, Alexandra Papoutsaki, Benjamin J. Raphael, Eli Upfal

**Affiliations:** 1 Department of Mathematics and Computer Science, University of Southern Denmark, Funen, Denmark; 2 Department of Computer Science, Brown University, Providence, Rhode Island, United States of America; 3 Center for Computational Molecular Biology, Brown University, Providence, Rhode Island, United States of America; Ontario Institute for Cancer Research, CANADA

## Abstract

A key challenge in genomics is to identify genetic variants that distinguish patients with different *survival time* following diagnosis or treatment. While the log-rank test is widely used for this purpose, nearly all implementations of the log-rank test rely on an asymptotic approximation that is not appropriate in many genomics applications. This is because: the two populations determined by a genetic variant may have very different sizes; and the evaluation of many possible variants demands highly accurate computation of very small *p*-values. We demonstrate this problem for cancer genomics data where the standard log-rank test leads to many false positive associations between somatic mutations and survival time. We develop and analyze a novel algorithm, Exact Log-rank Test (ExaLT), that accurately computes the *p*-value of the log-rank statistic under an exact distribution that is appropriate for any size populations. We demonstrate the advantages of ExaLT on data from published cancer genomics studies, finding significant differences from the reported *p*-values. We analyze somatic mutations in six cancer types from The Cancer Genome Atlas (TCGA), finding mutations with known association to survival as well as several novel associations. In contrast, standard implementations of the log-rank test report dozens-hundreds of likely false positive associations as more significant than these known associations.

## Author Summary

The identification of genetic variants associated with survival time is crucial in genomic studies. To this end, a number of methods have been proposed to computing a *p*-value that summarized the difference in survival time of two or more population. The most widely used method among these is the log-rank test. Widely used implementations of the log-rank test present a systematic error that emerges in most genome-wide applications, where the two populations have very different sizes, and the accurate computation of very small *p*-values is required due to the evaluation of a number of candidate variants. Considering cancer genomic applications, we show that the systematic error leads to many false positive associations of somatic variants and survival time. We present and analyze a new algorithm, ExaLT that accurately computes the *p*-value for the log-rank test under a distribution that is appropriate for the parameters found in genomics. Unlike previous approaches, ExaLT allows to control the accuracy of the computation. We use ExaLT to analyze cancer genomics data from The Cancer Genome Atlas (TCGA), identifying several novel associations in addition to well known associations. In contrast, the standard implementations of the log-rank test report a huge number of presumably false positive associations.

## Introduction

Next-generation DNA sequencing technologies are now enabling the measurement of exomes, genomes, and mRNA expression in many samples. The next challenge is to interpret these large quantities of DNA and RNA sequence data. In many human and cancer genomics studies, a major goal is to find associations between an observed phenotype and a particular variable (e.g., a single nucleotide polymorphism (SNP), somatic mutation, or gene expression) from genome-wide measurements of many such variables. For example, many cancer sequencing studies aim to find somatic mutations that distinguish patients with fast-growing tumors that require aggressive treatment from patients with better prognosis. Similarly, many human disease studies aim to find genetic alleles that distinguish patients who respond to particular treatments, i.e. live longer. In both of these examples one tests the association between a DNA sequence variant and the *survival time*, or length of time that patients live following diagnosis or treatment.

**Fig 1:**
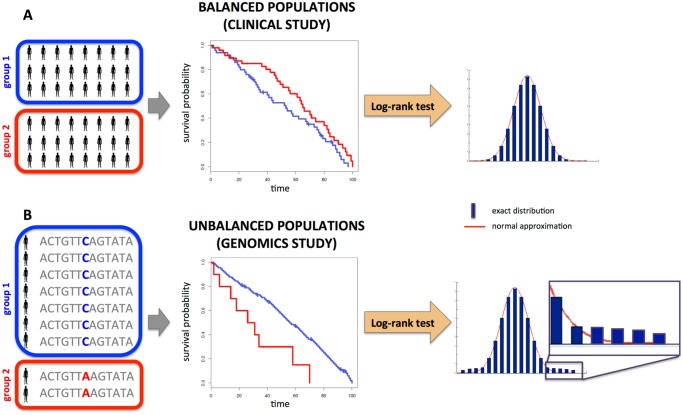
Difference between survival analysis in a clinical setting with balanced populations and genomics setting, with unbalanced populations. (A) In a typical clinical study, two pre-selected groups of similar size are compared. Because the groups are balanced and each has a suitable number of patients, the asymptotic approximation (normal distribution) used in common implementations of the log-rank test gives an accurate approximation of the exact distribution, resulting in accurate *p*-values. (B) In a genomics study, the two groups are defined by a genetic variant. In many cases, the sizes of the groups are unbalanced, with one group being much larger than the other. In this situation, the asymptotic distribution does not accurately approximate the exact distribution of the log-rank statistic, and the resulting *p*-values computed from the tail of the distribution (see inset) are inaccurate.

The most widely approach to determine the statistical significance of an observed difference in survival time between two groups is the log-rank test [1, 2]. An important feature of this test, and related tests in survival analysis [3], is their handling of *censored* data: in clinical studies, patients may leave the study prematurely or the study may end before the deaths of all patients. Thus, a lower bound on the survival time of these patients is known. Importantly, many studies are designed to test survival differences between two pre-selected populations that differ by one characteristic; e.g. a clinical trial of the effectiveness of a drug. These populations are selected to be approximately equal in size with a suitable number of patients to achieve appropriate statistical power (Fig 1A). In this setting, the null distribution of the (normalized) log-rank statistic is asymptotically (standard) normal; i.e. follows the (standard) normal distribution in the limit of infinite sample size. Thus, nearly every available implementation (e.g., the LIFETEST procedure in SAS, and the survdiff R function, and coin and exactRankTests packages in R and SPlus) of the log-rank test computes *p*-values from the normal distribution, an approximation that is accurate asymptotically (see S1 Text).

The design of a genomics study is typically very different from the traditional clinical trials setting. In a genomics study, high-throughput measurement of many genomics features (e.g. whole-genome sequence or gene expression) in a cohort of patients is performed, and the goal is to *discover* those features that distinguish survival time. Thus, the measured individuals are repeatedly partitioned into two populations determined by a genomic variable (e.g. a SNP) and the log-rank test, or related survival test, is performed (Fig 1B). Depending on the variable the sizes of the two populations may be very different: e.g. most somatic mutations identified in cancer sequencing studies, including those in driver genes, are present in < 20% of patients [4–9]. Unfortunately, in the setting of unbalanced populations, the normal approximation of the log-rank statistic might give poor results.

While this fact has been noted in the statistics literature [10–12], it is not widely known, and indeed the normal approximation to the log-rank test is routinely used to test the association of somatic mutations and survival time (e.g. [13, 14] and numerous other publications). A second issue in genomics setting is that the repeated application of the log-rank test demands the accurate calculation of very small *p*-values, as the computed *p*-value for a single test must be corrected for the large number of tests; e.g. through a Bonferroni or other multiple-hypothesis correction. An inaccurate approximation of *p*-values will result in an unacceptable number of false positives/negative associations of genomic features with survival. These defining characteristics of genomics applications, unbalanced populations and necessity of highly-accurate *p*-values for multiple-hypothesis correction, indicate that standard implementations of the log-rank test are inadequate.

We propose to compute the *p*-value for the log-rank test using an exact distribution determined by the observed number of individuals in each population. Perhaps the most famous use of an exact distribution is Fisher’s exact test for testing the independence of two categorical variables arranged in a 2×2 contingency table. When the counts in the cells of the table are small, the exact test is preferred to the asymptotic approximation given by the *χ* ^2^ test [15]. Exact tests for comparing two survival distributions have received scant attention in the literature. There are three major difficulties in developing such a test. First, there are multiple observed features that determine the exact distribution including the number of patients in each population and the observed censoring times. With so many combinations of parameters it is infeasible to pre-compute distribution tables for the test. Thus, we need an efficient algorithm that computes the *p*-value for any given combination of observed parameters. Second, we cannot apply a standard Monte-Carlo permutation test to this problem since we are interested in very small *p*-values that are expensive to accurately estimate with such an approach. (By the accurate estimation of a *p*-value using Monte-Carlo permutation test, we mean the calculation of a *p*-value and a confidence interval of the same order of magnitude of the *p*-value.) Third, there are two possible null distributions for the log-rank test, the conditional and the permutational [2, 16–18]. While both of these distributions are asymptotically normal, the permutational distribution is more appropriate for genomics settings [2, 19], as we detail below. Yet no efficient algorithm is known to compute the *p*-value of the log-rank test under the exact permutational distribution.

We introduce an efficient and mathematically sound algorithm, called ExaLT (for Exact Log-rank Test), for computing the *p*-value under the exact permutational distribution. (ExaLT computes an estimate of the *p*-value under the *exact* permutational distribution; for this reason we denote the *p*-value obtained from ExaLT as an *exact *p*-value*, in contrast to the *approximate *p*-value* obtained from asymptotic distributions.) The run-time of ExaLT is not function of the *p*-value, enabling the accurate calculation of small *p*-values. For example, obtaining an accurate estimate of *p* ≈ 10^−9^ is required if one wants to test the association of 1% of the human genome (e.g., the exome) with survival, and using a standard MC approach requires (with the Clopper-Pearson confidence interval estimate) the evaluation of ≥ 10^11^ samples, that for a population of 200 patients requires > 8 days; in contrast ExaLT is capable of estimating *p* ≈ 10^−13^ on 200 patients in < 2 hours. In contrast to heuristic approaches (see Materials and Methods) ExaLT provides rigorous guarantees on the relation between the estimated *p*-value and the correct *p*-value; moreover, it returns a conservative estimate of the *p*-value, thus guaranteeing rigorous control on the number of false discoveries. We test ExaLT on data from two published cancer studies [20, 21], finding substantial differences between the *p*-values obtained by our exact test and the approximate *p*-values obtained by standard tools in survival analysis. In addition, we run ExaLT on somatic mutation and survival data from The Cancer Genome Atlas (TCGA) and find a number of mutations with significant association with survival time. Some of these such as *IDH1* mutations in glioblastoma are widely known; for others such as *BRCA2* and *NCOA3* mutations in ovarian cancer there is some evidence in the literature; while the remaining are genuinely novel. Most of these are identified only using the exact permutational test of ExaLT. In contrast, the genes reported as highly significant using standard implementations of the log-rank test are not supported by biological evidence; moreover, these methods report dozens-hundreds of such likely false positive associations as more significant than known genes associated with survival. These results show that our algorithm is practical, efficient, and avoids a number of false positives, while allowing the identification of genes known to be associated with survival and the discovery of novel, potentially prognostic biomarkers.

## Results

### Accuracy of Asymptotic Approximations

We first assessed the accuracy of the asymptotic approximation for the log-rank test on simulated data from a cohort of 500 patients with a gene *g* mutated in 5% of these patients, a frequency that is not unusual for cancer genes in large-scale sequencing studies [4–7]. We compared the survival times of the population 𝓟(*g*) of patients with a mutation in *g* to the survival of the population $\bar{𝓟}(g)$ of patients with no mutation in *g*. We computed *p*-values using R survdiff on multiple random instances (in order to obtain a distribution for the *p*-value of *g*) in which 𝓟(*g*) and $\bar{𝓟}(g)$ have the same survival distribution. S1 Fig shows that the *p*-values computed by the asymptotic approximation are much smaller than expected under the null hypothesis, with the smallest *p*-values showing the largest deviation from the expected uniform distribution.

**Fig 2:**
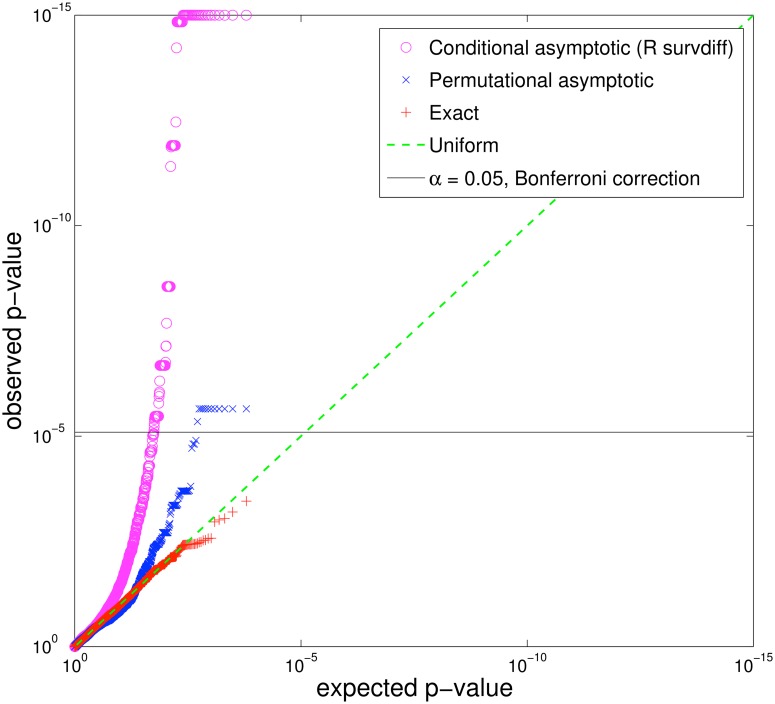
Differences between observed and expected *p*-values from different forms of the log-rank test on a randomized cancer dataset consisting of somatic mutations in 6184 genes. The *p*-values for the genes should be distributed uniformly (green line), since there is no association between mutations and survival in this random data. Asymptotic approximations of the log-rank statistic (purple and blue) yield *p*-values that deviate significantly from the uniform distribution, incorrectly reporting many genes whose mutations are significantly associated with survival. In particular, the asymptotic log-rank test in R reports 110 genes with significant association, using a Bonferroni corrected *p*-value < 0.05 (black line), or 291 genes with significant association using a less conservative FDR = 0.05. In contrast, the exact test makes no false discoveries.

The inaccuracy of the asymptotic log-rank test results in a large number of false discoveries: for example, considering a randomized version of a cancer mutation dataset (S1 Table) in which no mutation is associated with survival (i.e. no true positives), the asymptotic log-rank test reports 110 false discoveries (Bonferroni correction) or 291 false discoveries (False Discovery Rate (FDR) correction), with significance level *α* = 0.05 (Fig 2). We found that for the number of patients of interest to current genomic studies, the inaccuracy of the asymptotic log-rank test results mostly from the imbalance in the sizes of the two populations, rather than the total number of patients or the number of patients in the smaller population (see S1a Fig, S1b Fig, S1c Fig, S1d Fig, and S1 Text).

### An Efficient Algorithm for the Exact Log-Rank Test

As noted above, there are two exact distributions for the log-rank test in the literature: the permutational distribution [2] and the conditional distribution [16]. We developed an algorithm, Exact Log-rank Test (ExaLT), to compute the *p*-value of the log-rank statistic under the exact permutational distribution. On simulations of cancer data, we found that the *p*-values from the permutational exact test are significantly closer to the empirical *p*-values than the *p*-values obtained from the conditional exact test (S2 Fig and S1 Text). Thus, we derived a fully polynomial time approximation scheme (FPTAS) for computing the *p*-value under the permutational distribution. In contrast to heuristic methods that do not provide any rigorous guarantee on the quality of the approximation of the *p*-value, our algorithm provides an approximation that is guaranteed to be within a user defined distance from the *p*-value, for any given sample size, in polynomial time. Furthermore, the output of our scheme is always a conservative or valid *p*-value estimate. The C++ implementation of ExaLT (that can be called from R) is available at https://github.com/fvandin/ExaLT.

### Published Cancer Studies

To demonstrate the applicability of ExaLT we compared the *p*-values from the exact distribution to *p*-values from the asymptotic approximation reported in two recently published cancer genomics studies [20, 21]. Huang et al. [20] divides patients into groups defined by the number of risk alleles of five single nucleotide polymorphisms (SNPs), and compares the survival distribution of the resulting populations. In one comparison, the survival distribution of 2 patients (13% of total) with at most 2 risk alleles was compared with the survival distribution of 14 patients with more than 2 risk alleles, and a *p*-value of 0.012 is reported. Thus, this association is significant at the traditional significance level of *α* = 0.05. However, ExaLT computes an exact *p*-value of 0.17, raising doubts about this association. In another comparison patients at a different disease stage were considered, and the division of the patients into groups as above resulted in comparing the survival distribution of 8 patients (17% of total) with the survival distribution of 40 patients, and a *p*-value of 6×10^−6^ is reported. In contrast, ExaLT computes an exact *p*-value of 2×10^−3^, a reduction of three orders of magnitude in the significance level. Additional comparisons are shown in the S1 Text. Therefore in these cases the asymptotic approximation underestimates the exact permutational *p*-values resulting in associations deemed more significant than what is supported by the data.

In [21], the survival distribution of 14 glioblastoma patients (11% of total) with somatic *IDH1* or *IDH2* mutations was compared to the survival distribution of 115 patients with wild-type *IDH1* and *IDH2*. The reported *p*-value from the asymptotic approximation is 2×10^−3^, while the exact permutational *p*-value is 5×10^−4^, indicating a *stronger* association between somatic mutations in *IDH1* or *IDH2* and (longer) survival than reported. Notably, this same association has been reported in three other glioblastoma studies [22–24].

### TCGA Cancer Data

**Fig 3:**
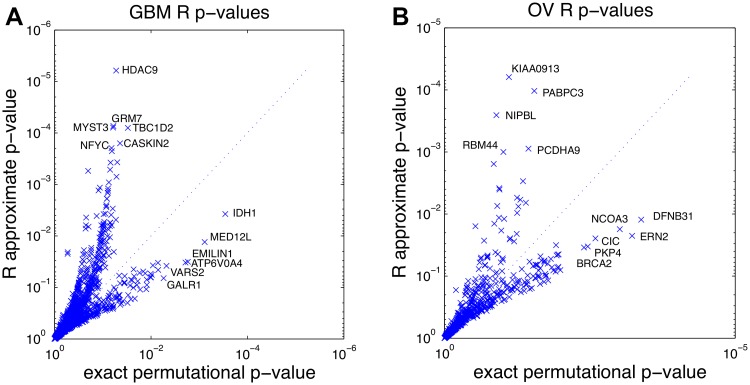
Comparison of the *p*-values of association between somatic mutations and survival time for TCGA glioblastoma (GBM) and ovarian (OV) datasets. Each data point represents a gene. (A) Comparison of the R survdiff *p*-values and the exact permutational *p*-values for the GBM dataset. (B) Comparison of the R survdiff *p*-values and the exact permutational *p*-values for the OV dataset.

We analyzed somatic mutation and survival data from studies of six different cancer types (S1 Table) from The Cancer Genome Atlas (TCGA). For the range of parameters of these datasets our simulations show that the asymptotic approximation is not accurate for genes with mutation frequency ≤ 10%; we then did not considered genes with mutation frequency > 10%. We also discarded genes mutated with frequency < 1%. For each mutated gene, we first obtained an estimate $\tilde{p}$ of the *p*-value using an MC approach, and if $\tilde{p}\leq 0.01$ we used ExaLT to compute a controlled approximation of the *p*-value. We compared the *p*-value obtained in this way with the one obtained by using the asymptotic approximation as computed by the R package survdiff (S2 Table, S3 Table, S4 Table, S5 Table). Fig 3 shows the exact *p*-values and the R survdiff *p*-values for the glioblastoma multiforme (GBM) dataset and ovarian serous adenocarcinoma (OV) dataset. The *p*-values for the other datasets are shown in S3 Fig.

For most datasets the asymptotic *p*-values obtained from R survdiff are very different from the ones obtained with the exact *p*-values obtained by ExaLT, and the ranking of the genes by *p*-value is very different as well (see S1 Text). For example, in GBM *none* of the top 26 genes reported by R are in the list of the top 26 genes reported by the exact permutational test. Since genomics studies are typically focused on the *discovery* of novel hypotheses that will be further validated, this striking difference in the ranking of genes by the two algorithms is important: a poor ranking of genes by their association with survival will lead to many false discoveries undergoing additional experimental validation. While some of genes ranked in the top 10 by ExaLT are known to have mutations associated with survival (e.g., *IDH1* in GBM and *BRCA2* in OV), *none* of the top 10 genes reported by R survdiff (S5 Table) have mutations known to be associated with survival. R survdiff ranks dozens-hundreds of presumably false positives associations as more significant than these known genes. Moreover, R survdiff reports extremely strong association with survival for many of these higher ranked, but likely false positive, genes; e.g., in uterine corpus endometrial carcinoma (UCEC), 13 genes have *p* < 10^−8^ and an additional 19 genes have *p* < 10^−5^, but none of these have a known association with survival.

The top 10 genes reported by ExaLT contain several novel associations that are supported by the literature and are not reported using R survdiff. In GBM, ExaLT identifies *IDH1* (*p* ≤ 3×10^−4^), *VARS2* (*p* ≤ 5×10^−3^) and *GALR1* (*p* ≤ 6×10^−3^), among others. As noted above, the association between mutations in *IDH1* and survival has been previously reported in GBM [21–24]. A germline variant in *VARS2* has been reported to be a prognostic marker, associated with survival, in early breast cancer patients [25]. The expression of *GALR1* has been reported to be associated with survival in colorectal cancer [26], and its inactivation by methylation has been associated with survival in head and neck cancer [27, 28]. In OV, ExaLT identifies *BRCA2* (*p* ≤ 4×10^−3^) and *NCOA3* (*p* ≤ 10^−3^), and others. Germline and somatic mutations in *BRCA2* (and *BRCA1*) have been associated with survival in two ovarian cancer studies [4, 29]. A polymorphism in *NCOA3* has been associated with breast cancer [30], and its amplification has been associated with survival in ER-positive tumors [31].

Thus, the exact test implemented by ExaLT appears to have higher sensitivity and specificity in detecting mutations associated with survival on the sizes of cohorts analyzed in TCGA. Finally, we note that the exact conditional test obtains results similar to R survdiff, confirming that the the exact permutational test implemented by ExaLT is a more appropriate exact test for genomics studies. (See S1 Text.) We have also assessed the difference between the result obtained using ExaLT and the results obtained using the asymptotic permutational test (S2 Table, S3 Table, S4 Table, S5 Table). In this case the difference in the ranking of genes is reduced but still present; in particular, in COADREAD 4 of the top 10 genes identified by ExaLT are not among the top 10 genes found using the asymptotic permutational test. Moreover, there are some genes for which there is a large difference in the *p*-value computed by ExaLT and the *p*-value from the asymptotic permutational test; for example, in UCEC data *CTGF* is ranked first by both ExaLT and the asymptotic permutational test, but it has *p* = 9.6×10^−5^ by ExaLT and *p* = 8.3×10^−10^ by the asymptotic permutational test.

## Discussion

In this work we focus on the problem of performing survival analysis in a genomics setting, where the populations being compared are not defined in advance, but rather are determined by a genomic measurement. The two distinguishing features of such studies are that the populations are typically unbalanced and that many survival tests are performed for different measurements, requiring highly accurate *p*-values for multiple hypothesis testing corrections. We show empirically that the asymptotic approximations used in available implementations of the log-rank test produce anti-conservative estimates of the true *p*-values when applied to unbalanced populations, resulting in a large number of false discoveries. This is not purely a phenomenon of small population size: the approximation remains inaccurate even for a large number of samples (e.g., 100) in the small population. This inaccuracy makes asymptotic approximations unsuitable for cancer genomic studies, where the vast majority of the genes are mutated in a small proportion of all samples [4–6] and also for genome-wide association studies (GWAS) where rare variants may be responsible for a difference in drug response or other phenotype, even if it is possible that for extremely large genomic studies (e.g., with 100000 patients) the asymptotic approximations would provide results accurate enough even for imbalanced populations (e.g., when the small population is 1%).

The problem with the log-rank test for unbalanced populations has previously been reported [10, 11], but the implications for genomics studies have not received attention. Note that the issue of unbalanced populations is further exacerbated by any further subdivision of the data: e.g. by considering mutations in specific locations or protein domains; by considering the impact of mutations on a specific therapeutic regimen; by testing the association of mutations with survival in a particular subtype of cancer; by grouping into more than two populations; or by correcting for additional covariates such as age, stage, grade, etc. All of these situations occur in genomics studies.

We considered the two versions of the log-rank test, the conditional [16] and the permutational [2], and we found that the exact permutational distribution is more accurate in genomics settings. We introduce ExaLT, the first efficient algorithm to compute highly accurate *p*-values for the exact permutational distribution. We implemented and tested our algorithm on data from two published cancer studies, showing that the exact permutational *p*-values are significantly different from the *p*-values obtained using the asymptotic approximations. We also ran ExaLT on somatic mutation and survival data from six cancer types from The Cancer Genome Atlas (TCGA), showing that our algorithm is practical, efficient, and allows the identification of genes known to be associated with survival in these cancer types as well as novel associations. We note that ExaLT can be employed as part of permutation tests that require the computation of *p*-values for a large number of genomic features measured on the same set of patients [12]. While the current implementation of ExaLT handles ties in survival times by breaking them arbitrarily, its extension to different tie breaking strategies and their impact is an important future direction.

The method we propose can be generalized to assess the difference in survival between more than two groups, by considering the exact permutational distribution for the appropriate test statistic. For this reason, our method can be adapted to test the difference in survival between groups of patients that have homozygous or heterozygous mutations, or to test whether the presence of a group of genomic features has a different effect on survival compared to the presence of the single genomic features. For the same reason, our method can incorporate categorical covariates, while it is unclear how methods based on the log-rank test, as ours, can incorporate continuous covariates or how they can be used to assess specific (e.g., additive) models of interactions between genomic features and survival.

While our focus here was the log-rank test, our results are relevant to more general survival statistics. First, in some survival analysis applications, samples are given different weights; our algorithm can be easily adapted to a number of these different weighting schemes. Second, an alternative approach in survival analysis is to use the Cox Proportional-Hazards model [3]. While in the Cox regression model one can easily adjust for categorical and continuous covariates, it is not clear how to incorporate continuos potential confounders in the log-rank test that we consider. While this constitutes a limitation of our method, the Cox regression models is often used to compare two populations even when no adjustment for confounders is performed. In this case, the significance of the resulting coefficients in the regression is typically done using a test that is equivalent to the log-rank test, and thus our results are relevant for this approach as well. See S1 Text and S4 Fig.

The challenges of extending multivariate regression models to the multiple-hypothesis setting of genome-wide measurements is not straightforward. Direct application of such a multivariate Cox regression will often not give reasonable results as: there are a limited number of samples and a large number of genomic variants; and many variants are rare and not associated with survival. Witten and Tibshirani (2010) [32] recently noted these difficulties for gene expression data stating that: “*While there are a great number of methods in the literature for identification of significant genes in a microarray experiment with a two-class outcome … the topic of identification of significant genes with a survival outcome is still relatively unexplored.*” We propose that exact tests such as the one provided here will be useful building blocks for more advanced models of survival analysis in the genomics setting.

## Materials and Methods

### Background: The Log-Rank Test

We focus here on the two-sample log-rank test of comparing the survival distribution of two groups, *P*_0_ and *P*_1_. Let *t*_1_ < *t*_2_ < … < *t*_*k*_ be the times of observed, uncensored events; in case of ties, we assume that they are broken arbitrarily. Let *R*_*j*_ be the number of patients *at risk* at time *t*_*j*_, i.e. the number of patients that survived (and were not censored) up to this time, and let *R*_*j*,1_ be the number of *P*_1_ patients at risk at that time. Let *O*_*j*_ be the number of observed uncensored events in the interval (*t*_*j*−1_, *t*_*j*_], and let *O*_*j*,1_ be the number of these events in group *P*_1_. If the survival distributions of *P*_0_ and *P*_1_ are the same, then the expected value $E[O_{j,1}]=O_{j}\frac{R_{j,1}}{R_{j}}$. The log-rank statistic [1, 2] measures the sum of the deviations of *O*_*j*,1_ from the expectation, $V=\sum_{j=1}^{k}\left(O_{j,1}-O_{j}\frac{R_{j,1}}{R_{j}}\right)$.

(In some clinical applications one is more interested in either earlier or later events. In that case the statistic is a weighted sum of the deviations. Our results easily translate to the weighted version of the test.) Under the null hypothesis of no difference in the survival distributions of the two groups, *E*[*V*] = 0, and *Pr*(∣*V*∣ ≥ ∣*v*∣) is the *p*-value of an observed value *v*. Two possible null distributions are considered in the literature, the permutational distribution and the conditional distribution (see S5 Fig).

### Permutational Log-Rank Test

In the *permutational* log-rank test [2], the null distribution is obtained by assigning each patient to population *P*_0_ or *P*_1_ independently of the survival time. Let *n* be the total number of patients, and *n*_1_ the number of patients in group *P*_1_. We consider the sample space of all $\left(\begin{array}{c} n\\ n_{1} \end{array}\right)$ possible selections of survival times and censoring information from the observed data for the *n*_1_ patients of group *P*_1_. Each such selection is assigned equal probability ${\left(\begin{array}{c} n\\ n_{1} \end{array}\right)}^{-1}$.

### Conditional Log-Rank Test

In the conditional log-rank test [16], the null distribution is defined by conditioning on *O*_*j*_, *R*_*j*_, and *R*_*j*,1_ for *j* = 1,…, *k*. If at time *t*_*j*_ there are a total of *R*_*j*_ patients at risk, including *R*_*j*,1_ patients in *P*_1_, then under the assumption of no difference in the survival of *P*_0_ and *P*_1_ the *O*_*j*_ events at time *t*_*j*_ are split between *P*_0_ and *P*_1_ according to a hypergeometric distribution with parameters *R*_*j*_, *R*_*j*,1_, and *O*_*j*_.

### Choice of Null Distribution and Estimation of *p*-values

We considered the two versions of the log-rank test, the conditional [16] and the permutational [2], that differ in the null distribution they consider. The conditional log-rank test is preferred in clinical trials because it does not assume equal distribution of censoring in the two populations. This is important in clinical trials when patients in the two groups are subject to different treatments that may affect their probability of leaving the trial. However, unequal censoring is not a concern in genomic studies, since we do not expect a DNA sequence variant to have an impact of the censoring in the population. Moreover, in the genomic studies of interest to this work, the patients are not assigned to the two populations at the beginning of the study, and the measured individuals are instead repeatedly partitioned into two populations determined by a genomic variable. Therefore under the null hypothesis of no association between a genomic variable and survival time the two populations can be assumed to have the same distribution of potential follow-up times, and the correction for unequal follow-up, that is a concern in clinical trials [33], is not required in our scenario. In both versions of the test, under the null distribution the prefix sums of the log-rank statistic define a martingale, and by the martingale central limit theorem [3], the normalized log-rank statistic has an asymptotic 𝓝(0,1) distribution. (Sometimes the log-rank test is described using an asymptotic *χ* ^2^ distribution; the two version of the tests are related, and our results hold for the version of the log-rank test based *χ* ^2^ distribution as well (S1 Fig).) The normalizing variance is different in the two null distributions and this may be reflected in differences between the *p*-values obtained from the two null distributions, but asymptotically the two variances are the same [17], leading to the same *p*-values in the two versions of the test for large balanced populations. Therefore, the distinction between the two versions of the test is largely ignored in practice, where most papers that use the log-rank test or software packages that implement the test do not document the specific version test they consider. This can be explained, in part, by the widespread use of the log-rank in other scenarios, like clinical trials, where the issues specific to genomic settings (e.g., the imbalance between populations) do not arise. The differences between the tests are also rarely discussed in the literature, although there is some discussion [17, 19] on which variance is the appropriate one to use to compute *p*-values from the asymptotic approximation.

In the case of small and unbalanced populations, the two null distributions yield different *p*-values, and the normal approximation gives poor estimates of both (S1 Fig). On simulations of cancer data, we found that the *p*-values from the permutational exact test are closer to the empirical *p*-values than the *p*-values obtained from the conditional exact test (S2 Fig and S1 Text). Moreover, we prefer the permutational null distribution because it better models the null hypothesis for mutation data.

While the exact computation of *p*-values in the conditional null distribution can be computed in polynomial time using a dynamic programming algorithm [33], no polynomial time algorithm is known for the exact computation of the *p*-value in the permutational null distribution: current implementations are based on a complete enumeration algorithm, making its use impractical for large number of patients (e.g., the StatXact manual recommends using the enumeration algorithm only when the number of samples is at most 20). Several heuristics have been developed for related computations including: saddlepoint methods to approximate the mid-*p*-values [34], methods based on the Fast Fourier Transform (FFT) [35–37], and branch and bound methods [38]. Such heuristics are shown to be *asymptotically* correct, converging to the correct *p*-value as the number of samples and computation time grows to infinity. However, no explicit bounds are known for the accuracy of the computed *p*-value when these heuristics are applied to a fixed sample size and under a bounded computation time. Therefore, when run on a specific input, these heuristics do not provide guarantees on the relation between the *p*-value and the approximation they report. Given the systematic error we report below for the standard asymptotic implementation of the log-rank test, we argue that such guarantees are essential in this and many similar settings.

### ExaLT

We developed an algorithm, Exact Log-rank Test (ExaLT), to compute the *p*-value of the log-rank statistic under the exact permutational distribution. In particular, we designed a fully polynomial time approximation scheme (FPTAS) for computing the *p*-value under the permutational distribution. Our algorithm gives an explicit bound on the error in approximating the true *p*-value, for any given sample size, in polynomial time. Furthermore, the output of our scheme is always a conservative or valid *p*-value estimate. Conceptually, our algorithm is similar to the one presented in [39].

Since the log-rank statistic depends only on the *order* of the events and not on their actual times, we can without loss of generality treat the survival data (including the censored times) as an ordered sequence of events, with no two patients having identical survival times. Let *n*_*j*_ = ∣*P*_*j*_∣, for *j* = 0,1, be the number of patients in each population and let *n* = *n*_0_+*n*_1_ be the total number of patients. We represent the data by two binary vectors **x** ∈ {0,1}^*n*^ and **c** ∈ {0,1}^*n*^, where *x*_*i*_ = 1 if the *i*th event was in *P*_1_ and *x*_*i*_ = 0 otherwise; *c*_*i*_ = 0 if the *i*th event was censored and *c*_*i*_ = 1 otherwise. Note that $n_{1}=\sum_{i=1}^{n}x_{i}$. In this notation the log-rank statistic is

$$\begin{array}{c} V=V\left(x,c\right)=\sum_{j=1}^{n}c_{j}(x_{j}-\frac{n_{1}-\sum_{i=0}^{j-1}x_{i}}{n-j+1})\cdot \end{array}$$ (1)

Let $V_{t}(\text{x})=\sum_{j=1}^{t}c_{j}(x_{j}-\frac{n_{1}-\sum_{i=0}^{j-1}x_{i}}{n-j+1})$ be the test statistic *V*(**x**) at time *t*. Note that since *n*, *n*_1_, and **c** are fixed, the statistic depends only on the value of **x**. Assume the observed log-rank statistic has value *v*. The *p*-value of the observation *v* is the probability *Pr*(∣*V*(**x**)∣ ≥ ∣*v*∣) computed in the probability space in which the *n*_1_ events of *P*_1_ are uniformly distributed among the *n* events.

For any 0 ≤ *t* ≤ *n* and 0 ≤ *r* ≤ *n*_1_, let $P(t,r,v)=Pr(V_{t}(\text{x})\leq v\text{and}\;\sum_{i=1}^{t}x_{i}=r)$ denote the joint probability of *V*_*t*_(**x**) ≤ *v* and exactly *r* events from *P*_1_ occur in the first *t* events. Let $Q(t,r,v)=Pr(V_{t}(\text{x})\geq v\text{and}\;\sum_{i=1}^{t}x_{i}=r)\cdot$ denote the joint probability of *V*_*t*_(**x**) ≥ *v* and exactly *r* events from *P*_1_ occur in the first *t* events.

At time 0: *P*(0,*r*,*v*) = 1 if *r* = 0 and *v* ≥ 0, otherwise *P*(0,*r*,*v*) = 0. Similarly *Q*(0,*r*,*v*) = 1 if *r* = 0 and *v* ≤ 0, otherwise *Q*(0,*r*,*v*) = 0.

Given the values of *P*(*t*,*r*,*v*) for all *v* and *r*, we can compute the values of *P*(*t*+1,*r*,*v*) using the following relations: If *c*_*t*+1_ = 1 then

$$\begin{array}{c} \begin{array}{ccc} P(t+1,r,v) & = & \left(1-\frac{n_{1}-r}{n-t}\right)P\left(t,r,v+\frac{n_{1}-r}{n-t}\right)+\frac{n_{1}-\left(r-1\right)}{n-t}P\left(t,r-1,v-\left(1-\frac{n_{1}-\left(r-1\right)}{n-t}\right)\right)\cdot \end{array} \end{array}$$

If *c*_*t*+1_ = 0 then

$$\begin{array}{c} P\left(t+1,r,v\right)=\left(1-\frac{n_{1}-r}{n-t}\right)P\left(t,r,v\right)+\frac{n_{1}-\left(r-1\right)}{n-t}P\left(t,r-1,v\right)\cdot \end{array}$$

Analogous equations hold for *Q*(*t*,*r*,*v*).

The process defined by these equations guarantees that the *n* events always include *n*_1_ events of *P*_1_. Thus, the *p*-value of the observation *v* is given by *Pr*(∣*V*(**x**)∣ ≥ ∣*v*∣) = *P*(*n*,*n*_1_,−∣*v*∣)+*Q*(*n*,*n*_1_,∣*v*∣).

For fixed *t* and *r*, *P*(*t*+1,*r*,*v*) and *Q*(*t*+1,*r*,*v*) are step functions. For example, if *c*_*t*+1_ = 1, then as we vary *v*, *P*(*t*+1,*r*,*v*) changes only at the points in which $P\left(t,r-1,v-\left(1-\frac{n_{1}-(r-1)}{n-t}\right)\right)$ or $P\left(t,r,v+\frac{n_{1}-r}{n-t}\right)$ change values. Thus, we only need to compute the function *P*(*t*+1,*r*,*v*) at these points. At *t* = 0 the function *P*(0,*r*,*v*) assumes up to 2 values. If *P*(*t*,*r*,*v*) assumes *m*(*t*,*r*) values and *P*(*t*,*r*−1,*v*) assumes *m*(*t*,*r*−1) values, then *P*(*t*+1,*r*,*v*) assumes up to *m*(*t*,*r*)+*m*(*t*,*r*−1) values.

Similar relations hold for *P*(*t*+1,*r*,*v*) when *c*_*t*+1_ = 0, and for computing *Q*(*t*,*r*,*v*) in the two cases. Thus, in *n* iterations the process computes the exact probabilities *P*(*n*,*r*,*v*) and *Q*(*n*,*r*,*v*), but it may have to compute probabilities for an exponential number of different values of *v* in some iterations.

We construct a polynomial time algorithm by modifying the above procedure to compute the probabilities of only a polynomial number of values in each iteration. We first observe that since the probability space consists of $\left(\begin{array}{c} n\\ n_{1} \end{array}\right)$ equal probability events, all non-zero probabilities in our analysis are $\geq n^{-n_{1}}$. For *ɛ* > 0, fix *ɛ*_1_ such that (1−*ɛ*_1_)^−*n*^ = 1+*ɛ*. Note that *ε*_1_ = *O*(*ε*/*n*). We discretize the interval of possible non-zero probabilities $\left[n^{-n_{1}},1\right]$, using the values (1−*ɛ*_1_)^*k*^, for *k* = 0,…,ℓ, where $\ell =\frac{-n_{1}\log n}{\log(1-{ɛ}_{1})}=O({ɛ}^{-1}nn_{1}\log n)$. The approximation algorithm estimates *P*(*t*,*r*,*v*) with a step function $\tilde{P}(t,r,v)$ defined by a sequence of ℓ points $v_{k,r}^{t}$, *k* = 0,…,ℓ, such that $v_{k,r}^{t}$ is an estimate for the largest *v* such that *P*(*t*,*r*,*v*) ≤ (1−*ɛ*_1_)^*k*^. We prove that if iteration *t* computes a function $\tilde{P}(t,r,v){\left(1-\varepsilon_{1}\right)}^{t}\leq P(t,r,v)\leq \tilde{P}(t,r,v)$, then starting from $\tilde{P}(t,r,v)$ the *t*+1 iteration computes an estimate $\tilde{P}(t+1,r,v){\left(1-\varepsilon_{1}\right)}^{t+1}\leq P(t+1,r,v)\leq \tilde{P}(t+1,r,v)$. (S6 Fig provides the intuition for how the approximation at time *t*+1 is computed from the approximation at time *t*.) Thus, after *n* iterations we have an *ε*-approximations for *P*(*n*,*n*_1_,*v*). Similar computations obtain an *ε*-approximation for *Q*(*n*,*n*_1_,*v*). The details of the algorithm and analysis are given in the S1 Text.

We implemented the FPTAS in our software ExaLT and evaluated its performance as *n* and *ɛ* varies, and by comparing its running time with the running time of the exhaustive enumeration algorithm for the permutational test (S7 Fig). Our implementation of the FPTAS is very efficient, with significant speed-up compared to the exhaustive algorithm.

We note that, given parameters *n*,*n*_1_,*ɛ*, and given the censoring vector **c**, ExaLT computes the *entire distribution* of the test statistic under the null hypothesis. Once this distribution is computed, it can be used for *any* vector **x** ∈ {0,1}^*n*^ with $n_{1}=\sum_{i=1}^{n}x_{i}$ and the same censoring vector **c**. We therefore implemented a variant of ExaLT that given multiple vectors **x**’s for the same set of patients (e.g., mutation data from multiple genes on the same set of patients) as input stores the distributions already computed and uses these distributions for quick lookup whenever possible. (This feature cannot be used when the vectors **x**’s are defined for different sets of patients, for example when there are missing gene measurements or genotypes.)

### Synthetic Data

We used synthetic data to assess the accuracy of the asymptotic approximations. We generated data as follow: when no censoring was included, we generated the survival times for the patients from an exponential distribution, and the group labeling (mutated or not) were assigned to patients independently of their survival time; when censoring in *c*% of the patients was included, the survival times come from the exponential distribution with expectation equal to 30, and censoring variable from an exponential distribution resulting in *c*% of censoring (in expectation).

We used synthetic data to compare the empirical *p*-value and the *p*-values from the exact tests as well. In this case we generated synthetic data using two related but different procedures. In the first procedure, we mutate a gene *g* in exactly a fraction *f* of all patients. In the second procedure, we mutated a gene *g* in each patient independently with probability *f*. The second procedure models the fact that mutations in a gene *g* are found in each patient independently with a certain probability. In both cases the survival information is generated from the same distribution for all patients, as described above.

### Mutation and Clinical TCGA Data

We analyzed somatic mutation and clinical data, including survival information, from the public TCGA data portal (https://tcga-data.nci.nih.gov/tcga/). In particular we considered single nucleotide variants and small indels for colorectal carcinoma (COADREAD), glioblastoma multiforme (GBM), kidney renal clear cell carcinoma (KIRC), lung squamous cell carcinoma (LUSC), ovarian serous adenocarcinoma (OV), and uterine corpus endometrial carcinoma (UCEC). We restricted our analysis to patients for which somatic mutation and survival data were both available. We only considered genes mutated in > 1% of patients. We also removed genes with mutation frequency > 10%. Since genes mutated in the same set of patients would have the same association to survival, they are all equivalent for an automated analysis of association between mutations and survival; we then collapsed them into *metagenes*, recording the genes that appear in a metagene. For the remaining genes we first obtained an estimate $\tilde{p}$ of the *p*-value using a MC approach, and we then used ExaLT (with *ɛ* = 1.5) to compute the exact permutational *p*-value whenever $\tilde{p}$ was ≤ 0.01. For any given TCGA dataset we used the variant of ExaLT that stores previously computed distribution and uses them for quick lookup whenever possible. The runtime was reasonable for the dataset we analysed (e.g., 310 minutes for the COADREAD dataset).

The results published here are in whole or part based upon data generated by The Cancer Genome Atlas pilot project established by the NCI and NHGRI. Information about TCGA and the investigators and institutions who constitute the TCGA research network can be found at http://cancergenome.nih.gov/.

## Supporting Information

S1 Text Supporting Text. (PDF)

S1 Table Parameters of the cancer datasets analyzed. (PDF)

S2 Table The 10 genes with smallest *p*-values identified using the exact permutational test in cancer data. (PDF)

S3 Table The 10 genes with smallest *p*-values identified using the asymptotic permutational test in cancer data. (PDF)

S4 Table The 10 genes with smallest *p*-values identified using the exact conditional test in cancer data. (PDF)

S5 Table The 10 genes with smallest *p*-values identified using the asymptotic conditional test in cancer data. (PDF)

S1 Fig Comparison of the *p*-values from asymptotic approximations and the uniform distribution. (a) Distribution of *p*-values obtained using the conditional approximation, and distribution of *p*-values for the uniform distribution. Generated considering 10^5^ instances with *n*_1_ = 100 samples in the small population, different number *n* of samples in total, and same survival distribution for all patients (≈ 40% censoring). (b) Distribution of *p*-values obtained using the permutational approximation, and distribution of *p*-values for the uniform distribution. Generated considering 10^5^ data points with *n*_1_ = 5%*n* samples in the small population, *n* total samples, and no censoring. (c) Distribution of *p*-values obtained using the permutational approximation, and distribution of *p*-values for the uniform distribution. Generated considering 10^5^ data points with *n* = 100 total samples, different values of *n*_1_, and no censoring. (d) Distribution of *p*-values obtained using different approximations, and distribution of *p*-values for the uniform distribution. Generated considering 10^5^ instances with *n* = 500 total samples, *n*_1_ = 5%*n* samples with a mutations in the gene, and same survival distribution for all patients (≈ 40% censoring). (e) Distribution of *p*-values obtained using surv_test method in coin R package, and distribution of *p*-values for the uniform distribution. Generated considering 10^5^ instances with *n* = 500 total samples, *n*_1_ = 5%*n* samples with a mutations in the gene, and same survival distribution for all patients, with 0% or 40% censoring. (f) Distribution of *p*-values obtained using different approximations, and distribution of *p*-values for the uniform distribution. Generated considering 10^5^ instances with *n* = 500 total samples, *n*_1_ = 5%*n* samples with a mutations in the gene, and same survival distribution for all patients ( ≈ 60% censoring).
(PDF)

S2 Fig Comparison of the *p*-values from the exact tests and the empirical *p*-values for two different null distributions. The R coefficients comparing the −*log*_10_ exact *p*-values to the −*log*_10_ empirical *p*-values are the following: in Fig. (a), permutational = 0.96, conditional = 0.88; in Fig (b), permutational = 0.72, conditional = 0.43. (a) Comparison of exact conditional *p*-values, exact permutational *p*-values, and empirical *p*-values for *n* = 100,*n*_1_ = 5%*n*, and 30% censoring. Each point represents an instance of survival data. (b) Comparison of exact conditional *p*-values, exact permutational *p*-values, and empirical *p*-values for *n* = 100, expectation *n*_1_ = 5%*n*, and 30% censoring. Each point represents an instance of survival data.
(PDF)

S3 Fig Comparison of log-rank *p*-values on cancer data. Comparison of the *p*-values from the exact permutational test, the exact conditional test, the asymptotic conditional approximation (as implemented in the survdiff function in R), and the asymptotic permutational approximation for cancer datasets COADREAD, GBM, KIRC, LUSC, OV, UCEC. (a,b,c,d,e,f): Each data point represents a gene, and the *p*-values computed using the exact permutational test and the *p*-values from R survdiff for the gene are shown. (g,h,i,j,k,l): Each data point represents a gene, and the *p*-values computed using the exact permutational test and the exact conditional test for the gene are shown. (m,n,o,p,q,r): Each data point represents a gene, and the *p*-values computed using the exact permutational test and the asymptotic permutational test for the gene are shown.
(PDF)

S4 Fig Cox Proportional-Hazard *p*-values. Comparison of the *p*-values from asymptotic approximations for the Cox Proportional-Hazard model and the uniform distribution, and comparison of the *p*-values from exact permutational tests and the Cox likelihood ratio test with the empirical *p*-values for two different null distributions. (a) Distribution of *p*-values obtained using the asymptotic approximation for the Cox Proportional-Hazard model and the distribution of *p*-values for the uniform distribution. Generated considering 9×10^5^ instances with *n* = 200 total samples, *n*_1_ = 5 samples in the small population and same survival distribution for all patients (no censoring). (b) Comparison of Cox likelihood ratio *p*-values, exact permutational *p*-values, and empirical *p*-values for *n* = 100,*n*_1_ = 5%*n*, and 30% censoring. Each point represents an instance of survival data. (c) Comparison of Cox likelihood ratio *p*-values, exact permutational *p*-values, and empirical *p*-values for *n* = 100, expectation(*n*_1_) = 5%*n*, and 30% censoring. Each point represents an instance of mutations and survival data. The R coefficients comparing the −*log*_10_ exact *p*-values to the −*log*_10_ empirical *p*-values are the following: in Fig. (b), permutational = 0.96, Cox likelihood ratio = 0.70; in Fig (c), permutational = 0.72, Cox likelihood ratio = 0.46.
(PDF)

S5 Fig Survival data, and log-rank test. (Top) The log-rank test compares the Kaplan-Meier curves of the two groups. (Middle) Survival data is represented by sorting patients by increasing survival. **x** represents group labels for patients, and **c** represents censoring information (*c*_*i*_ = 0 if event at time *t*_*i*_ is censored, *c*_*i*_ = 1 otherwise). (Bottom) The conditional test is defined by a series of independent contingency tables with marginals corresponding to the number of patients at risk in each group and the number of events in each group, conditioning on the patients at risk at each non-censored time; *O*_*i*_ denotes the number of events at time *t*_*i*_, *R*_*i*,*j*_ denotes the number of patients at risk in group *j* at time *i*. The permutational test considers all $\left(\begin{array}{c} n\\ n_{1} \end{array}\right)$ possible locations of the *n*_1_ patients with label 1 in the vector **x**.
(PDF)

S6 Fig Summary of FPTAS. Starting from the approximation $\tilde{P}(t,r,v)$ at time *t* that uses ℓ values of *v* to approximate *P*(*t*,*r*,*v*), in the *t*+1 iteration the FPTAS computes an approximation $\hat{P}(t+1,r,v)$ for *P*(*t*+1,*r*,*v*) that uses up to 2ℓ values of *v*; then the approximation $\tilde{P}(t+1,r,v)$ is built starting from $\hat{P}(t+1,r,v)$ by appropriately reducing the number of values of *v* considered, while maintaining guarantees on the approximation.
(PDF)

S7 Fig Running time of the FPTAS, and comparison with the running time of the exhaustive enumeration algorithm. (a) Runtime of FPTAS and of the exhaustive enumeration for different values of *n*, and for *n*_1_ = 10,*ɛ* = 5, no censoring. (b) Runtime of FPTAS and of the exhaustive enumeration for *n* = 100,*ɛ* = 5, no censoring, and different values of *n*_1_. (c) Runtime of the FPTAS for different values of *ɛ*, and for *n* = 100,*n*_1_ = 10, no censoring. (d) Comparison of the FPTAS *p*-values and the exact *p*-values (obtained with the complete enumeration algorithm) for *n* = 60,*n*_1_ = 4, no censoring, and *ɛ* = 1.5.
(PDF)
