# Supplementary material for: Accurate Computation of Survival Statistics in Genome-Wide Studies
Source: PLoS Comput Biol. 2015 May 7;11(5):e1004071. doi: 10.1371/journal.pcbi.1004071 (PMC4423942; doi:10.1371/journal.pcbi.1004071)
Supplement: S1 Text — (PDF) [file pcbi.1004071.s001.pdf]

Supporting Text for "Accurate Computation of Survival Statistics in Genome-wide Studies"

Fabio Vandin[1,2,3], Alexandra Papoutsaki[2,3], Benjamin J. Raphael[2,3,*], and Eli Upfal[2,*]

[1]Department of Mathematics and Computer Science, University of Southern Denmark, DK.
[2]Department of Computer Science, Brown University, USA.
[3]Center for Computational Molecular Biology, Brown University, USA.
[*]Corresponding authors.

Implementations of the log-rank test

Common statistical packages provide implementations[1] of the log-rank test for the following distributions:

- asymptotic conditional: SAS (`LIFETEST`), R and S-Plus (`survdiff`), SPSS, GraphPad Prism.
- asymptotic permutational and exact permutational: StatXact, R (`surv_test`).

Background

Model

Suppose a set $\mathcal{G}$ of genes was sequenced in a collection $\mathcal{P}$ of patients, all of whom have the same disease. Each sequenced gene[2] $g \in \mathcal{G}$ partitions the set of patients into two subsets: the $\mathcal{P}(g)$, with patients with a mutation in g, and the $\bar{\mathcal{P}}(g)$, with patients with no mutation in g. The goal is to identify genes whose mutational status is highly correlated with the survival time, in the sense that the survival distribution of patients in $\mathcal{P}(g)$ is different from the survival distribution of patients in $\bar{\mathcal{P}}(g)$. A key challenge in survival analysis is dealing with *censored* patients whose exact survival time is unknown. Censoring occurs for a variety of reasons, but the most common is that the study only lasts for a finite amount of time, and some fraction of patients remain alive at the conclusion of the study. In addition, during the course of the study patients may leave the study for a variety of reasons, that are unrelated to their treatment or disease state. The censored survival time is the last time the patient was observed in the study, which is a lower bound for the patient's survival time[3]. Survival analysis assumes that censoring is *non informative*, i.e. the event that a patient is censored is independent of the patient's survival beyond the censoring time. The log-rank test [1] (or family of tests) is the most commonly used non-parametric test for comparing the survival distribution of two or more populations with data subject to censoring. The advantage of this test is that it includes the censored data in its statistic, rather then removing it from the data. Since a large fraction of patients may be censored (e.g., up to 94% in the data below), it is not desirable to remove this "missing data" from consideration. In the section below, we describe two different versions of the log-rank test, the conditional log-rank and the permutational log-rank test.

[1]This information was derived directly from the software manual and/or the publication cited in the manual.

[2]One may also consider mutations at different levels of resolution; e.g. partitioning patients according to mutations in individual nucleotides or protein domains.

[3]In some references patients who survived the study are called *right censored* and patients who withdrew from the study are called *randomly* censored.

Basic survival analysis: the log-rank test

We focus on the two-samples log-rank test of comparing the survival distribution of two groups, P_0 and P_1. Let $t_1 < t_2 < \cdots < t_k$ be the times of observed not censored events. Let R_j be the number of patients *at risk* at time t_j, i.e. the number of patients that survived (and were not censored) up to this time, and let $R_{j,1}$ be the number of P_1 patients at risk at that time. Let O_j be the number of observed, not censored events in the interval $(t_{j-1}, t_j]$, and let $O_{j,1}$ be the number of these events in group P_1. If the survival distributions of P_0 and P_1 are the same then in expectation $E[O_{j,1}] = O_j \frac{R_{j,1}}{R_j}$. The log-rank statistic [1, 2] measures the sum of the deviations of $O_{j,1}$ from this equal distribution[4] expectation,

$$V = \sum_{j=1}^{k} \left(O_{j,1} - O_j \frac{R_{j,1}}{R_j} \right). \tag{1}$$

Since the log-rank statistic depends only on the *order* of the events and not on their actual times, we can w.l.o.g. treat the survival data (including censored times) as an ordered sequence of events, with no two patients having identical survival times. Let $n_i = |P_i|$ be the number of patients in each set and let $n = n_0 + n_1$ be the total number of patients. We represent the data with two binary vectors $\mathbf{x} \in \{0,1\}^n$ and $\mathbf{c} \in \{0,1\}^n$, where $x_i = 1$ if the ith event was in P_1 and $x_i = 0$ otherwise; $c_i = 0$ if the ith event was censored and $c_i = 1$ otherwise. Note that $n_1 = \sum_{i=1}^{n} x_i$. In this notation the log-rank statistic is

$$V = V(\mathbf{x}, \mathbf{c}) = \sum_{j=1}^{n} c_j \left(x_j - \frac{n_1 - \sum_{i=1}^{j-1} x_i}{n - j + 1} \right). \tag{2}$$

Clearly, the further V is from zero, the more likely it is the case that the two survival distributions are different. To quantify this intuition, we define the null hypothesis of no difference in the survival distributions of the two groups, and then compute the distribution of the test statistic V under the null hypothesis. Two possible null distributions are considered in the literature, defining two versions of the log-rank test (S5 Fig.).

Conditional log-rank test [16]. In this version, the null distribution is defined by conditioning on O_j and $R_{j,1}$ for $j = 1, \ldots, k$. If at time t_j there are a total of R_j patients at risk, including $R_{j,1}$ patients in P_1, then under the assumption of no difference in the distributions of P_0 and P_1, we expect the O_j events at that time to be split between P_0 and P_1 according to a hypergeometric distribution with parameters R_j, $R_{j,1}$, and O_j.

Under this null distribution the expectation of the log-rank statistic is 0, and its variance is $\sigma_h^2 = \sum_{j=1}^{k} O_j \frac{R_{1,j}}{R_j} \left(1 - \frac{R_{1,j}}{R_j}\right) \frac{R_j - O_j}{R_j - 1}$ (Mantel-Haenszel variance [16]).

Note that this test does not assume equal distribution of censoring in the two groups. This property is important in clinical trials when patients in the two groups are subject to different treatments that may affect their probability of leaving the trial. In the case of cancer mutation data, under the null hypothesis it is unlikely that the presence of a mutation changes the probability that a patient leaves the trial, and thus we do not face this difficulty. However, a major disadvantage of this test is that the number of events in P_1, generated by this distribution, is a random variable that is equal to n_1 only on average. In the case of an unbalanced population, where n_1 is small, it can significantly affect the computed p-value.

Permutational log-rank test [2]. In this version, we observe that under the null hypothesis the distribution of the group labels, x_i's, is independent of the survival information. Therefore, we consider the sample space of all $\binom{n}{n_1}$ possible locations of the n_1 patients of group P_1 in the vector $\mathbf{x}$, and each possibility is

[4] In some clinical applications one is more interested in either earlier or later events. In that case, the statistic is a weighted sum of the deviations. Our results easily translate to the weighted version of the test.

assigned equal probability $\binom{n}{n_1}^{-1}$. For this reason, the resulting distribution is usually called *permutational* distribution of the log-rank statistic [2]. Under this null hypothesis the expectation of the log-rank statistic is 0, and the variance [19] is $\sigma_p^2 = \frac{n_1 n_2}{n(n-1)} \left(k - \sum_{i=1}^{k} \frac{1}{R_i} \right)$. Note that in this distribution the number of patients in P_1 is exactly n_1. The validity of this log-rank test depends on the probability of censoring being equal in the two groups. As discussed above this assumption holds in our application.

Estimating the p-value

Under both null distributions above, the expectation $E[V] = 0$. Given an observed value v, its p-value is $Pr(|V| \geq |v|)$. In the two null distributions the prefix sums of the log-rank statistic define a martingale, and therefore, by the martingale central limit theorem [3], the normalized log-rank statistic V/σ, where σ is either σ_h or σ_p, has an asymptotic $\mathcal{N}(0,1)$ distribution, which gives an easy method for computing the p-value. Furthermore, asymptotically the two variances σ_h^2 and σ_p^2 are the same [17], thus for large balanced populations the two versions of the test give the same results. Therefore, the distinction between the two versions of the test is mostly ignored in the literature, although there is some discussion of which variance is the appropriate to use [17, 19].

The situation is drastically different in the setting of genome-wide cancer survival analysis. As was reported in [10, 11] and we show in the next section, the normal approximation gives a poor estimate for the p-value in the range of population sizes inherent in the genome-wide association studies. Thus, we need an efficient algorithm for computing a correct estimate of the p-values that does not depend on the Normal approximation. Furthermore, we also report that in this range of parameters, the p-value of the log-rank statistic is very sensitive to the choice of null distribution: since the conditional distribution matches the problem parameters only in expectation, we prefer the permutational null distribution that matches exactly the problem's parameters.

Accuracy of Asymptotic Approximations

We applied the log-rank test based on asymptotic approximations to randomly generated survival and mutation data. We focused on the case of unbalanced populations. We compared the p-values obtained from the asymptotic approximations with the uniform distribution that is expected under the null hypothesis. We use n to denote the total number of samples, and n_1 the number of samples in the small population. S1a Fig. shows that even when the number of patients in the small population is large ($n_1 = 100$), when the imbalance between populations increases, the accuracy of the asymptotic approximation decreases. S1b Fig. shows that for a fixed ratio n_1/n, the asymptotic approximation improves when the total populations size increases. S1c Fig. shows that for a fixed n, the asymptotic approximation improves when the imbalance decreases. In addition to the normal approximation, S1d Fig. includes the χ^2 approximation, and shows the results considering 10^5 data points with $n = 500$ total samples, $n_1 = 5\%n$ samples with a mutation in the gene, and same survival distribution for all patients. In particular, the survival time comes from an exponential distribution with the same expectation (equal to 30), and censoring variable from an exponential distribution resulting in 40% of censoring. These results show that with $n = 100$, n_1 must be $> 20\%n$ for the asymptotic permutational approximation to be accurate, while with $n = 500$, n_1 must be $\geq 5\%$ for the asymptotic permutational approximation to be accurate. We also used the method `surv_test` from the `R` package `coin` to compute asymptotic p-values. We tested the case of $n = 500, n_1 = 1\%n$, and 0% or 40% censoring. The resulting distribution of p-values (S1e Fig.) is consistent with the results obtained in the simulations above. We also repeated the experiment of S1d Fig. with 60% censoring, obtaining similar results (S1f Fig.).

Comparison of Exact Tests on Synthetic Data

Comparison of Exact Distributions

We find that the p-values from the permutational exact test are significantly closer ($p < 10^{-3}$) to the empirical p-values than the p-values obtained from the conditional exact test (S2 Fig.).

We compared the accuracy of exact p-values for the permutational and conditional distributions in our setting of unbalanced small populations using synthetic data. We generate synthetic data using two related but different procedures. In the first procedure, we mutate a gene g in exactly a fraction f of all patients. In the second procedure, we mutated a gene g in each patient independently with probability f. The second procedure models the fact that mutations in a gene g are found in each patient independently with a certain probability (that depends on the background mutation rate, the length of the gene, etc.). Thus, when repeating a study on a cohort of patients of the same size only the expected number of patients in which g is mutated is the same, and the observed number may vary. In both cases the survival information is generated from the same distribution for all patients. The survival time comes from the exponential distribution with expectation equal to 30, and censoring variable from an exponential distribution resulting in 40% of censoring in expectation. In S2a Fig. we compare the p-values computed from the exact permutational test and the exact conditional test with the empirical p-values for the first distribution, while in S2b Fig. we compare the p-values computed from the exact permutational test and the exact conditional test with the empirical p-values for the second distribution. In particular, we generated 10000 random input instances according to the first or the second distribution, and for each random input instance we computed the empirical p-values by using 10000 random instances generated (from the corresponding distribution) independently for each p-value and also computed the p-values from the two exact tests. In both cases the p-values (restricted to p-values ≤ 0.01) from the exact permutational distribution have a higher R coefficient than the p-values from the exact conditional distribution when compared to the empirical p-values (considering the $-log_{10}$ p-values in order to compute the R coefficient). Therefore the p-values from the permutational exact test are closer to the empirical p-values than the p-values obtained from the conditional exact test.

Algorithms

As shown by the results in previous sections, to carry out an effective genome-wide survival analysis for cancer somatic mutation we need an accurate estimate of the log-rank statistic p-values in the permutational null distribution.

While the exact p-value in the conditional test can be computed in quadratic time [42, 33] no polynomial time algorithm is known for the problem of computing the exact p-value for the permutational test. Abd-Elfattah and Butler [34] use saddlepoint methods to determine the mid-p-values for the permutational distribution. Heuristic methods may be derived from solutions to related problems. In particular the method of Pagano and Tritchler [37], based on the Fast Fourier Transform (FFT), may be adapted to compute some approximation of the exact p-value in polynomial time, but no guarantee on the accuracy of the approximation is provided by their method. Branch and bound methods (in the spirit of the method proposed by Bejerano et al. [38]) may be used to compute the exact p-value, but may require exponential time in the worst case. Note that since p-values can be really small, we do not want to use an MCMC approach, that requires to sample a number of random permutations at least proportional to c^{-1} in order to obtain an estimate for a p-value equal to c.

In the permutational distribution n and n_1 are fixed, and thus computing the p-value is equivalent to solving the following counting problem.

More Extreme Assignments Counting Problem: *Given $n, n_1 \in \mathbb{N}$, with $n_1 \leq n$, $v \in \mathbb{R}$ and $\mathbf{c} \in \{0,1\}^n$ determine the number of vectors $\mathbf{x} \in \{0,1\}^n$ that satisfy: $\sum_{i=1}^{n} x_i = n_1$ and $|V(\mathbf{x}, \mathbf{c})| \geq v$.*

Dividing the number of vectors $\mathbf{x}$ by $\binom{n}{n_1}$, which defines the sample space size, gives the p-value of v.

Based on the similarity between this problem and Knapsack Counting Problem [41], we conjecture that the problem may also be $\#P$-complete.

FPTAS for the permutational distribution

We provide a Fully Polynomial Time Approximation Scheme (FPTAS) to estimate the p-value from the permutational distribution, i.e. an algorithm that given $n, n_1, \mathbf{c}$, and v, for any $\varepsilon > 0$ computes an ε-approximation of $Pr(|V(\mathbf{x})| \geq v)$, that is a value p with $Pr(|V(\mathbf{x})| \geq v) \leq p \leq (1+\varepsilon)Pr(|V(\mathbf{x})| \geq v)$ in time that is polynomial in n and ε^{-1}. The FPTAS is derived from a pair of recurrence relations that compute the exact probability, but may not terminate in polynomial time. We then modify the process to obtain a fully polynomial time approximation scheme.

Exact computation. Let $V_t(\mathbf{x}) = \sum_{j=1}^{t} c_j \left(x_j - \frac{n_1 - \sum_{i=0}^{j-1} x_i}{n-j+1} \right)$ be the test statistic $V(\mathbf{x})$ at time t. Note that since n, n_1, and $\mathbf{c}$ are fixed, the statistic depends only on the values of $\mathbf{x}$. Assume the observed log-rank statistic has value v. The p-value of the observation v is the probability $Pr(|V(\mathbf{x})| \geq |v|)$ computed in the probability space in which the n_1 events of P_1 are uniformly distributed among the n events. For any $0 \leq t \leq n$ and $0 \leq r \leq n_1$, let $P(t, r, v)$ denote the joint probability $V_t(\mathbf{x}) \leq v$ and exactly r events of P_1 in the first t events,

$$P(t, r, v) = \Pr\left(V_t(\mathbf{x}) \leq v \text{ AND } \sum_{i=1}^{t} x_i = r \right).$$

Similarly, let $Q(t, r, v)$ denote the joint probability of $V_t(\mathbf{x}) \geq v$ and exactly r events of P_1 in the first t steps,

$$Q(t, r, v) = \Pr\left(V_t(\mathbf{x}) \geq v \text{ AND } \sum_{i=1}^{t} x_i = r \right).$$

At time 0,

$$P(0, r, v) = \begin{cases} 1 & \text{if } r = 0 \text{ and } v \geq 0 \\ 0 & \text{otherwise,} \end{cases} \quad \text{and} \quad Q(0, r, v) = \begin{cases} 1 & \text{if } r = 0 \text{ and } v \leq 0 \\ 0 & \text{otherwise.} \end{cases}$$

Given the values of $P(t, r, v)$ and $Q(t, r, v)$ for all v and r, we can compute the values of $P(t+1, r, v)$ and $Q(t, r, v)$ using the following relations:

If $c_{t+1} = 1$ then

$$P(t+1, r, v) = (1 - \frac{n_1 - r}{n-t})P(t, r, v + \frac{n_1 - r}{n-t}) + \frac{n_1 - (r-1)}{n-t}P(t, r-1, v - (1 - \frac{n_1 - (r-1)}{n-t})), \text{ and}$$

$$Q(t+1, r, v) = (1 - \frac{n_1 - r}{n-t})Q(t, r, v + \frac{n_1 - r}{n-t}) + \frac{n_1 - (r-1)}{n-t}Q(t, r-1, v - (1 - \frac{n_1 - (r-1)}{n-t})).$$

If $c_{t+1} = 0$ then

$$P(t+1, r, v) = (1 - \frac{n_1 - r}{n-t})P(t, r, v) + \frac{n_1 - (r-1)}{n-t}P(t, r-1, v), \text{ and}$$

$$Q(t+1, r, v) = (1 - \frac{n_1 - r}{n-t})Q(t, r, v) + \frac{n_1 - (r-1)}{n-t}Q(t, r-1, v).$$

The process defined by these equation guarantees that the n events include n_1 events of P_1. Thus, for $r \neq n_1$, $P(n, r, v) = 0$ and $Q(n, r, v) = 0$, and the p-value is given by[5]

$$Pr(|V(\mathbf{x})| \geq |v|) = P(n, n_1, -|v|) + Q(n, n_1, |v|).$$

[5]In the exact computation $Q(n, n_1, V) = 1 - P(n, n_1, V)$, but in the approximate algorithm below we need to compute each of the probability functions separately.

The functions $P(t+1,r,v)$ and $Q(t+1,r,v)$ are step functions. To compute the function $P(t+1,r,v)$ when $c_{t+1}=1$, by the definition of $P(t+1,r,v)$ we note that, for fixed r and t, its value changes only for values of v in which $P(t,r,v+\frac{n_1-r}{n-t})$ or $P(t,r-1,v-(1-\frac{n_1-(r-1)}{n-t}))$ change values. Thus, we need to compute the function $P(t+1,r,v)$ only for such values of v, that can be readily obtained from the values of v' for which $P(t,r,v')$ and $P(t,r-1,v')$ change value.

At $t=0$ the function $P(0,r,v)$ assumes up to 2 values. If $P(t,r,v)$ assumes $m(t,r)$ values and $P(t,r-1,v)$ assumes $m(t,r-1)$ values, then $P(t+1,r,v)$ assumes up to $m(t,r)+m(t,r-1)$ values. Similar relation hold for $P(t+1,r,v)$ when $c_{t+1}=0$, and for computing $Q(t,r,v)$ in the two cases. Thus, in n iterations the process computes the exact probabilities $P(n,r,v)$ and $Q(n,r,v)$, but it may have to compute probabilities for an exponential number of different values of v in some iterations.

Approximation Algorithm. We first note that since the probability space consists of $\binom{n}{n_1}$ equal probability events, all non-zero probabilities in our analysis are $\geq n^{-n_1}$. For $0<\varepsilon<1$, fix ε_1 such that $(1-\varepsilon_1)^{-n}=1+\varepsilon$. Note that $\epsilon_1=O(\epsilon/n)$. We discretize the interval of possible non-zero probabilities $[n^{-n_1},1]$, using the values $(1-\varepsilon_1)^k$, for $k=0,\ldots,\ell=\frac{-n_1\log n}{\log(1-\varepsilon_1)}=O(\varepsilon^{-1}nn_1\log n)$. The approximation algorithm computes estimates for $P(t,r,v)$ and $Q(t,r,v)$ in two separate processes.

Estimating $P(t,r,v)$. Let $\tilde{P}(t,r,v)$ be a step function defined by a sequence of points $v^t_{k,r}$, $k=0,\ldots,\ell$. The value of the function in the interval $(v^t_{k+1,r},v^t_{k,r}]$ is $(1-\varepsilon_1)^k$, for $v>v^t_{0,r}$ the value of the function is 1, and for $v<v^t_{\ell,r}$ the value of the function is 0. Consecutive points in the sequence may be the same ($v^t_{k+1,r}=v^t_{k,r}$), in that case the value of $\tilde{P}(t,r,v)$ is $(1-\varepsilon_1)^{k_v}$, where $k_v=\arg\max_k[v\leq v^t_{k,r}]$. (Note that the sequence $v^t_{k,r}$, $k=0,\ldots,\ell$ is non-increasing in k, since larger k corresponds to smaller probability.)

For $t=0$, we define $\tilde{P}(0,r,v)$ by the set of points $v^0_{k,0}=0$ and $v^0_{k,r}=\infty$ for $r>0$, $k=0,\ldots,\ell$. These functions satisfy $\tilde{P}(0,r,v)=P(0,r,v)$ for all r and v.

Assume that iteration $t+1$ starts with a set of functions $\tilde{P}(t,r,v)$, for $r=0,\ldots,n_1$ such that for all r and v

$$(1-\varepsilon_1)^t\tilde{P}(t,r,v)\leq P(t,r,v)\leq\tilde{P}(t,r,v).$$

We show that iteration $t+1$ computes functions $\tilde{P}(t+1,r,v)$ with the same approximation properties. (S6 Fig. shows how the approximation at time t + 1 is computed from the approximation at time t.)

To compute an estimate for the functions $P(t+1,r,v)$, $r=0,\ldots,n_1$, we use the relations given in the exact computation, estimating $P(t,r,v)$ by $\tilde{P}(t,r,v)$. In the case $c_{t+1}=1$ we use

$$\hat{P}(t+1,r,v)=(1-\frac{n_1-r}{n-t})\tilde{P}(t,r,v+\frac{n_1-r}{n-t})+\frac{n_1-(r-1)}{n-t}\tilde{P}(t,r-1,v-(1-\frac{n_1-(r-1)}{n-t})),$$

and compute (for each r) the function at the 2ℓ points corresponding to change in values in the functions $\tilde{P}(t,r,v)$ and $\tilde{P}(t,r-1,v)$:

$$v^t_{k,r}=v+\frac{n_1-r}{n-t}\ \text{ and } v^t_{k,r-1}=v-(1-\frac{n_1-(r-1)}{n-t}),\ \text{ for } k=1,\ldots,\ell.$$

In the case $c_{t+1}=0$ we use

$$\hat{P}(t+1,r,v)=(1-\frac{n_1-r}{n-t})\tilde{P}(t,r,v)+\frac{n_1-(r-1)}{n-t}\tilde{P}(t,r-1,v),$$

and compute the function (for each r) in the 2ℓ points $v^t_{k,r}$ and $v^t_{k,r-1}$.

Let $v_1\leq v_2\leq\cdots\leq v_{2\ell}$ be the 2ℓ points for which the value of $\hat{P}(t+1,r,v)$ was computed. We extend the function $\hat{P}(t+1,r,v)$ to a step function over all values of v, such that $\hat{P}(t+1,r,v)=\hat{P}(t+1,r,v_j)$, where j is the largest index such that $v\geq v_j$.

Since we computed the function $\hat{P}(t+1,r,v)$ in all points in which $\tilde{P}(t,r,v)$ and $\tilde{P}(t,r-1,v)$ change values, and by the assumptions on the values of $\tilde{P}(t,r,v)$, for all r and v we have

$$\hat{P}(t+1,r,v)(1-\varepsilon_1)^t \leq P(t+1,r,v) \leq \hat{P}(t+1,r,v).$$

We now approximate the function $\hat{P}(t+1,r,v)$ by a function $\tilde{P}(t+1,r,v)$ that is defined by the sequence of only ℓ values:

$$v_{k,r}^{t+1} = \arg\max_v [\hat{P}(t+1,r,v) \leq (1-\varepsilon_1)^k],\ k = 0,\dots,\ell.$$

Consider a value v such that $v_{k+1,r}^{t+1} \leq v < v_{k,r}^{t+1}$. We have:

$$P(t+1,r,v) \leq \hat{P}(t+1,r,v) \leq \hat{P}(t+1,r,v_{k,r}^{t+1}) \leq \tilde{P}(t+1,r,v_{k,r}^{t+1}) = \tilde{P}(t+1,r,v), \tag{3}$$

and

$$P(t+1,r,v) \geq \hat{P}(t+1,r,v)(1-\varepsilon_1)^t \geq \hat{P}(t+1,r,v_{k,r}^{t+1})(1-\varepsilon_1)(1-\varepsilon_1)^t \geq \tilde{P}(t+1,r,v)(1-\varepsilon_1)^{t+1}. \tag{4}$$

(The first inequality follows by the inductive assumption on $P(t+1,r,v)$ and $\hat{P}(t+1,r,v)$, the second inequality by the definition of v and $v_{k,r}^{t+1}$, and the third by the definition of $\tilde{P}(t+1,r,v)$.) Thus, our estimate $\tilde{P}(n,n_1,-v)$ for $P(V(x) \leq -v) = P(n,n_1,-v)$ satisfies

$$P(n,n_1,-v) \leq \tilde{P}(n,n_1,-v) \leq P(n,n_1,-v)\frac{1}{(1-\varepsilon_1)^n} \leq P(n,n_1,-v)(1+\varepsilon).$$

Estimating $Q(t,r,v)$. Recall that $Q(t,r,v)$ is the probability of exactly r events of P_1 in the first t steps and the statistic at time t is $\geq v$,

$$Q(t,r,v) = \Pr\left(\sum_{j=1}^{t} c_j\left(x_j - \frac{n_1 - \sum_{i=1}^{j} x_i}{n-j}\right) \geq v \text{ AND } \sum_{i=1}^{t} x_i = r\right).$$

Let $\tilde{Q}(t,r,v)$ be a step function defined by a sequence of points $v_{k,r}^t$, $k=0,\dots,\ell$. The value of the function in the interval $[v_{k,r}^t, v_{k+1,r}^t)$ is $(1-\varepsilon_1)^k$, for $v < v_{0,r}^t$ the value of the function is 1, and for $v > v_{\ell,r}^t$ the value of the function is 0. Consecutive points in the sequence may be the same ($v_{k+1,r}^t = v_{k,r}^t$), in that case the value of $\tilde{Q}(t,r,v)$ is $(1-\varepsilon_1)^{k_v}$, where $k_v = \arg\max_k[v \geq v_{k,r}^t]$. (Note that the sequence $v_{k,r}^t$, $k=0,\dots,\ell$ is monotone non-decreasing in k, since larger k corresponds to smaller probability.)

For $t=0$, we define $\tilde{Q}(0,r,v)$ by the set of points $v_{k,0}^0 = 0$ and $v_{k,r}^0 = -\infty$ for $r>0$, $k=0,\dots,\ell$. These functions satisfy $\tilde{Q}(0,r,v) = Q(0,r,v)$ for all r and v.

Assume that iteration $t+1$ starts with a set of functions $\tilde{Q}(t,r,v)$, for $r=0,\dots,n_1$ such that for all r and v

$$(1-\varepsilon_1)^t\tilde{Q}(t,r,v) \leq Q(t,r,v) \leq \tilde{Q}(t,r,v).$$

We show then iteration $t+1$ computes functions $\tilde{Q}(t+1,r,v)$ with the same approximation properties.

To compute an estimates for the functions $Q(t+1,r,v)$, $r=0,\dots,n_1$, we use the relations given in the exact computation, estimating $Q(t,r,v)$ by $\tilde{Q}(t,r,v)$. In the case $c_{t+1}=1$ we use

$$\hat{Q}(t+1,r,v) = (1 - \frac{n_1-r}{n-t})\tilde{Q}(t,r,v+\frac{n_1-r}{n-t}) + \frac{n_1-(r-1)}{n-t}\tilde{Q}(t,r-1,v-(1-\frac{n_1-(r-1)}{n-t})),$$

and compute (for each r) the function at the 2ℓ points corresponding to change in values in the functions $\tilde{Q}(t,r,v)$ and $\tilde{Q}(t,r-1,v)$:

$$v_{k,r}^t = v + \frac{n_1 - r}{n-t} \text{ and } v_{k,r-1}^t = v - (1 - \frac{n_1-(r-1)}{n-t}), \text{ for } k = 1,\dots,\ell.$$

In the case $c_{t+1} = 0$ we use

$$\hat{Q}(t+1,r,v) = (1 - \frac{n_1 - r}{n-t})\tilde{Q}(t,r,v) + \frac{n_1-(r-1)}{n-t}\tilde{Q}(t,r-1,v),$$

and compute the function (for each r) in the 2ℓ points $v_{k,r}^t$ and $v_{k,r-1}^t$.

Let $v_1 \le v_2 \le \cdots \le v_{2\ell}$ be the 2ℓ points for which the value of $\hat{Q}(t+1,r,v)$ was computed. We extend the function $\hat{Q}(t+1,r,v)$ to a step function over all values of v, such that $\hat{Q}(t+1,r,v) = \hat{Q}(t+1,r,v_j)$, where j is the largest index such that $v \ge v_j$.

Since we computed the function $\hat{Q}(t+1,r,v)$ in all points in which $\tilde{Q}(t,r,v)$ and $\tilde{Q}(t,r-1,v)$ change values, and by the assumptions on the values of $\tilde{Q}(t,r,v)$, for all r and v we have

$$\hat{Q}(t+1,r,v)(1-\varepsilon_1)^t \le Q(t+1,r,v) \le \hat{Q}(t+1,r,v).$$

We now approximate the function $\hat{Q}(t+1,r,v)$ by a function $\tilde{Q}(t+1,r,v)$ that is defined by the sequence of only ℓ values:

$$v_{k,r}^j = \arg\min_v[\tilde{Q}(j,r,v) \le (1-\varepsilon_1)^k], \; k = 0,\dots,\ell.$$

Consider a value v such that $v_{k,r}^{t+1} \le v < v_{k+1,r}^{t+1}$. We have:

$$Q(t+1,r,v) \le \hat{Q}(t+1,r,v) \le \hat{Q}(t+1,r,v_{k,r}^{t+1}) \le \tilde{Q}(t+1,r,v_{k,r}^{t+1}) = \tilde{Q}(t+1,r,v), \tag{5}$$

and

$$Q(t+1,r,v) \ge \hat{Q}(t+1,r,v)(1-\varepsilon_1)^t \ge \hat{Q}(t+1,r,v_{k,r}^{t+1})(1-\varepsilon_1)(1-\varepsilon_1)^t \ge \tilde{Q}(t+1,r,v)(1-\varepsilon_1)^{t+1}. \tag{6}$$

Thus, our estimate $\tilde{Q}(n,n_1,v)$ for $Q(V(x) \ge v) = Q(n,n_1,v)$ satisfies

$$Q(n,n_1,v) \le \tilde{Q}(n,n_1,v) \le Q(n,n_1,v)\frac{1}{(1-\varepsilon_1)^n} \le Q(n,n_1,v)(1+\varepsilon).$$

From the discussion above the following theorem is readily derived.

Theorem 1. *The algorithm above is a FPTAS for computing $Pr(|V(\mathbf{x})| \ge |v|)$.*

Proof. We first consider the approximation ratio. Note that $\Pr(|V(\mathbf{x})| \ge |v|) = \Pr(V(\mathbf{x}) \ge |v|) + \Pr(V(\mathbf{x}) \le -|v|)$; from the discussion above we have that $\Pr(V(\mathbf{x}) \le -|v|) \le \tilde{P}(n,n_1,-|v|) \le \Pr(V(\mathbf{x}) \le -|v|)(1+\varepsilon)$ and $\Pr(V(\mathbf{x}) \ge |v|) \le \tilde{Q}(n,n_1,|v|) \le \Pr(V(\mathbf{x}) \ge |v|)(1+\varepsilon)$, therefore if we define $\tilde{p} = \tilde{P}(n,n_1,-|v|) + \tilde{Q}(n,n_1,|v|)$ we have that $\Pr(|V(\mathbf{x})| \ge |v|) \le \tilde{p} \le (1+\varepsilon)\Pr(|V(\mathbf{x})| \ge |v|)$.

The run-time of each iteration is $O(\ell n_1) = O(\varepsilon^{-1} n n_1^2 \log n)$ and there are n iterations, thus for any $\varepsilon > 0$ the algorithm computes an ε-approximation in $O(\varepsilon^{-1} n^2 n_1^2 \log n)$ time. ◻

FPTAS running time

We ran experiments to study how the running time of the FTPAS varies for different values of n, n_1, and ε. We also compared the running time of the FPTAS with the running time of the exhaustive algorithm for permutational distribution. For simplicity, in our tests we assumed no censoring (i.e., $\mathbf{c} = 1^n$). Results are shown in S7 Fig. S7a Fig. shows the average runtime of the FPTAS with $n_1 = 10, \varepsilon = 5$, for different values of n. (Standard deviations are not shown since they are very small compared to the runtime.) For the same instances we also ran the exhaustive algorithm 10 times, stopping it after 5 hours (i.e., 18000 seconds) if it did not terminate. Results for the exhaustive algorithm are shown in S7a Fig. as well. The starred $(*)$ values of n are values for which the exhaustive algorithm was stopped after 5 hours in each of the 10 runs. The exhaustive algorithm is practical only for very small values of n, while the FPTAS can be used for much larger values of n. S7b Fig. shows how the runtime of the FPTAS varies for different values of n_1, with $n = 100, \varepsilon = 5$. As expected the runtime increases with n_1, but it is still practical for values of n_1 up to $0.2n$. We report the runtime of the exhaustive algorithm for comparison. Note that for $n = 100, n_1 = 20$ the exhaustive algorithm would take more than 160 years even running on a 100 Ghz machine under the unrealistic assumption that it could compute the log-rank statistic of a vector $\mathbf{x}$ every clock cycle. S7c Fig. shows how the runtime of the FPTAS varies for different values of the approximation parameter ε. We measured the runtime over 10 runs with $n = 100, n_1 = 10$, and no censoring. As expected, the runtime decreases by increasing ε. We also compared the p-value obtained using the FPTAS with the exact p-value (obtained with the complete enumeration algorithm) for $n = 60, n_1 = 4$, no censoring, and $\varepsilon = 1.5$. The results are shown in S7d Fig.

Cancer data

TCGA data

We analyzed somatic mutation and clinical data, including survival information, from the TCGA data portal (`https://tcga-data.nci.nih.gov/tcga/`). In particular we considered single nucleotide variants (SNVs) and small indels for colorectal carcinoma (COADREAD), glioblastoma multiforme (GBM), kidney renal clear cell carcinoma (KIRC), lung squamous cell carcinoma (LUSC), ovarian serous adenocarcinoma (OV), and uterine corpus endometrial carcinoma (UCEC). Since genes mutated in the same patients have the same association to survival, we collapsed them into *metagenes*, recording the genes that appear in a metagene. S1 Table shows a number of statistics for each dataset. We restricted our analysis to patients for which somatic mutation and survival data were both available. We only considered genes mutated in $> 1\%$ and in $< 10\%$ of patients. For each remaining gene, we first obtained an estimate $\tilde{p}$ of the p-value using an MC approach, and if $\tilde{p} \leq 0.01$ we used ExaLT to compute a controlled approximation of the p-value. S3 Fig. shows the comparison between the exact permutational p-value and the `R survdiff` p-value, the exact conditional p-value, or the asymptotic permutational p-value for each considered gene.

We compared the genes reported in the top positions by the different tests. The number of genes shared in the top positions of the lists obtained by the exact permutational test and the exact conditional test, and by the exact permutational test and `R survdiff` for the different cancer types are: in COADREAD, 8 genes are in the top 10 positions by the exact permutational test and by `R survdiff`, and 10 in the top 10 positions by the exact permutational test and by the exact conditional test, while 23 genes are in the top 25 positions by the exact permutational test and by `R survdiff`, and 23 in the top 25 positions by the exact permutational test and by the exact conditional test; in GBM, no gene is in the top 10 positions by the exact permutational test and by `R survdiff`, and 2 in the top 10 positions by the exact permutational test and by the exact conditional test, while no gene is in the top 25 positions by the exact permutational test and by `R` texttsurvdiff, and 2 in the top 25 positions by the exact permutational test and by the exact conditional test; in KIRC, 3 genes are in the top 10 positions by the exact permutational test and by `R survdiff`, and 4 in the top 10 positions by the exact permutational test and by the exact conditional test, while 10

genes are in the top 25 positions by the exact permutational test and by R `survdiff`, and 16 in the top 25 positions by the exact permutational test and by the exact conditional test; in LUSC, no gene is in the top 10 positions by the exact permutational test and by R `survdiff`, and no gene is in the top 10 positions by the exact permutational test and by the exact conditional test, while 1 gene is in the top 25 positions by the exact permutational test and by R `survdiff`, and 4 in the top 25 positions by the exact permutational test and by the exact conditional test; in OV, no gene is in the top 10 positions by the exact permutational test and by R `survdiff`, and 1 gene is in the top 10 positions by the exact permutational test and by the exact conditional test, while 3 genes are in the top 25 positions by the exact permutational test and by R `survdiff`, and 7 in the top 25 positions by the exact permutational test and by the exact conditional test; in UCEC, 5 genes are in the top 10 positions by the exact permutational test and by R `survdiff`, and 7 in the top 10 positions by the exact permutational test and by the exact conditional test, while 15 genes are in the top 25 positions by the exact permutational test and by R `survdiff`, and 24 in the top 25 positions by the exact permutational test and by the exact conditional test.

Published Cancer Studies

We analyzed differences between survival distributions reported in two published genomic studies [20, 21]. We considered only cases where the smallest population included at most 30% of all samples. We compared the exact permutational p-value with the p-value reported in the publications obtained using asymptotic approximations. Since the data for these studies is not publicly available, we inferred the data necessary to perform the log-rank test using the figures in the publications. In particular, since the exact time of events (censored or not) is not used by the log-rank test, we only inferred the order of events into the two populations and the censoring information. We then used R `survdiff` to obtain the p-value from the asymptotic approximation, and compared it with the p-value reported in the paper to validate the information we inferred from the figures.

In particular, for [20] we considered:

- Figure 2L: population sizes: 2 and 14. Reported $p = 0.012$; R `survdiff` $p = 0.012$; exact permutational $p = 0.17$;
- Figure 2I: population sizes: 8 and 48. Reported $p < 10^{-4}$; R `survdiff` $p = 6 \times 10^{-6}$; exact permutational $p = 2 \times 10^{-3}$;
- Figure 2H: population sizes: 9 and 21. Reported $p = 3.2 \times 10^{-3}$; R `survdiff` $p = 3.2 \times 10^{-3}$; exact permutational $p = 1.1 \times 10^{-2}$.

For [21] we considered:

- Figure 3A: population sizes: 14 and 115. Reported $p = 2 \times 10^{-3}$; R `survdiff` $p = 2.3 \times 10^{-3}$; exact permutational $p = 4.8 \times 10^{-4}$;
- Figure 3B: population sizes: 14 and 38. Reported $p < 10^{-3}$; R `survdiff` $p = 6 \times 10^{-6}$; exact permutational $p = 5 \times 10^{-4}$.

Comparison of Exact Permutational Test and Cox Proportional-Hazard Model on Synthetic Data

Three asymptotically equivalent statistical tests are commonly used to assess significance using the Cox Proportional-Hazard model: the score test, the Wald test, and the likelihood ratio test. All the three tests are based on an asymptotic approximation for the distribution of the test statistic.

We applied the three tests (score test, Wald test, and likelihood ratio test) to assess statistical significance under the Cox Proportional-Hazard Model, on randomly generated survival and mutation data, where

no mutation is associated with survival. The three tests are based on asymptotic approximations for the distribution of the test statistic. We focused on the case of unbalanced populations. In particular, we considered $n = 200$ total patients, and $n_1 = 5$ patients in the small population. We used the `R coxph` function to compute the p-values. S4a Fig. shows that for the score test and the Wald test the asymptotic approximation is inaccurate, while the asymptotic approximation is pretty accurate for the likelihood ratio test.

We then compared the accuracy of the p-values obtained with the exact permutational test and the p-values from the Cox likelihood ratio test. In particular, we use *synthetic data*, generated using the same distribution for the survival time and for the censoring time for all patients, and using different procedures to generate the mutation data. More specifically, we compared the empirical p-value (obtained by generating the data a number of times using the same parameters for the distribution) with the p-values from the exact permutational test and the p-values from the Cox likelihood ratio test using synthetic data. We generate the mutation data using two related but different procedures. In the first procedure, we mutate a gene g in exactly a fraction f of all patients. In the second procedure, we mutated a gene g in each patient independently with probability f. The second procedure models the fact that mutations in a gene g are found in each patient independently with a certain probability (that depends on the background mutation rate, the length of the gene, etc.). Thus, when repeating a study on a cohort of patients of the same size, only the expected number of patients in which g is mutated is the same, and the observed number may vary. In both cases, the survival information is generated from the same distribution for all patients. In particular, the survival time comes from the exponential distribution with expectation equal to 30, and censoring variable from an exponential distribution resulting in 30% of censoring. In S4b Fig. we compare the p-values computed from the exact permutational test and the Cox likelihood ratio test with the empirical p-values for the first distribution, while in S4c Fig. we compare the p-values computed from the exact permutational test and the Cox likelihood ratio test with the empirical p-values for the second distribution. In both cases we generated the empirical p-values as in S2 Fig.. In both cases, the p-values (restricted to p-values ≤ 0.01) from the exact permutational distribution have higher R coefficients than the p-values from the Cox likelihood ratio test when compared to the empirical p-values (considering the $-log_{10}$ p-values in order to compute the R coefficient).

The Cox proportional-hazards model is often used to correct for other variables that may be correlated to survival, like age, gender, or tumor stage. While the multivariate case is not the focus of this work, in this scenario a common rule of thumb [40, 43, 44] states that Cox models should be used with a minimum of 10 outcome events per predictor variable to obtain reliable results. This limits its applicability to moderately frequent events even in large genomic studies. For example, if we include three predictor variables in the model in addition to the mutation status of a gene, then only two of our seven cancer datasets (LUSC and UCEC) have more than 3 genes (7 and 8, respectively) that have the minimum recommended number of mutations. Extensions of the exact test presented here might prove useful in such settings of a small number of events per predictor variable. In particular, a stratified log-rank test using an exact distribution is a promising alternative.

References

[40] Concato J, Peduzzi P, Holford TR, and Feinstein AR. Importance of events per independent variable in proportional hazards analysis. i. background, goals, and general strategy. *J Clin Epidemiol*, 48(12):1495–501, Dec 1995.

[41] Dyer M, Frieze A, Kannan R, Kapoor A, Perkovic L, and Vazirani U. A mildly exponential time algorithm for approximating the number of solutions to a multidimensional knapsack problem. *Combinatorics, Probability and Computing*, 2(03):271–284, 1993.

[42] Mantel N, Patel NR, and Gray R. Computing an Exact Confidence Interval for the Common Odds Ratio in Several 2x2 Contingency Tables. *Journal of the American Statistical Association*, 80:969–973, 1985.

[43] Peduzzi P, Concato J, Feinstein AR, and Holford TR. Importance of events per independent variable in proportional hazards regression analysis. ii. accuracy and precision of regression estimates. *J Clin Epidemiol*, 48(12):1503–10, Dec 1995.

[44] Peduzzi P, Concato J, Kemper E, Holford TR, and Feinstein AR. A simulation study of the number of events per variable in logistic regression analysis. *J Clin Epidemiol*, 49(12):1373–9, Dec 1996.
