# Supplementary material for: Accurate Computation of Survival Statistics in Genome-Wide Studies
Source: PLoS Comput Biol. 2015 May 7;11(5):e1004071. doi: 10.1371/journal.pcbi.1004071 (PMC4423942; doi:10.1371/journal.pcbi.1004071)
Supplement: S1 Table — (PDF) [file pcbi.1004071.s002.pdf]

Table S1: Parameters of the cancer datasets analyzed.

dataset	num. patients	% censoring	num. mut. genes	num. genes: $1\% <$ mut. freq. $< 10\%$.
COADREAD	188	92%	2716	1090
GBM	268	30%	3702	1686
KIRC	292	74%	4448	1873
LUSC	175	60%	8465	5646
OV	315	43%	3740	664
UCEC	235	93%	8304	5770

For each dataset we show: the number of patients with both mutation and survival data; the percentage of patients with censored survival data; the number of (meta)genes mutated in at least one sample; the number of (meta)genes mutated in more than 1% and less than 10% of all samples.
