# Supplementary material for: Accurate Computation of Survival Statistics in Genome-Wide Studies
Source: PLoS Comput Biol. 2015 May 7;11(5):e1004071. doi: 10.1371/journal.pcbi.1004071 (PMC4423942; doi:10.1371/journal.pcbi.1004071)
Supplement: S3 Table — (PDF) [file pcbi.1004071.s004.pdf]

Table S3: The 10 genes with smallest p-values identified using the asymptotic permutational test in cancer data. For each cancer type, we show the top 10 genes, their rank, and p-value using the exact permutational test, the asymptotic permutational test, the exact conditional test, and `R survdiff`. The number of samples with a mutation in the gene is also reported.

		exact permutational		asymp. permutational		exact conditional		R `survdiff`		
cancer type	gene	rank	p	rank	p	rank	p	rank	p	num. mut.
COADREAD	PTPRM	1	5.5e-04	1	1.1e-05	4	2.3e-03	10	1.7e-05	8
	DOCK9	2	1.0e-03	2	4.6e-05	3	1.7e-03	5	4.3e-08	6
	DOCK3	6	4.2e-03	3	1.3e-04	8	1.1e-02	6	3.9e-06	3
	IGF1R	8	4.8e-03	4	1.5e-04	9	1.3e-02	7	8.9e-06	3
	ZNF568	10	5.4e-03	5	1.7e-04	10	1.5e-02	11	2.5e-05	3
	UBAP2L	13	6.4e-03	6	1.9e-04	15	1.8e-02	14	7.0e-05	3
	PCDHA12	12	6.1e-03	7	1.9e-04	14	1.8e-02	13	7.0e-05	3
	CASP8AP2	14	6.5e-03	8	2.0e-04	16	1.8e-02	16	7.8e-05	3
	VPS13B	3	1.6e-03	9	2.5e-04	1	3.6e-04	1	6.2e-12	8
	DOCK4	15	7.4e-03	10	2.5e-04	21	2.3e-02	24	2.7e-04	3
KIRC	CDCA2	1	2.8e-04	1	2.8e-04	1	1.8e-04	7	6.2e-10	6
	KIF27	4	1.8e-03	2	1.2e-03	13	6.2e-03	27	1.2e-04	4
	ATP10D	2	3.9e-04	3	1.2e-03	3	6.1e-04	5	3.6e-12	3
	TDRD7	3	9.1e-04	4	1.5e-03	5	1.5e-03	10	5.0e-09	3
	RELN	6	2.5e-03	5	1.8e-03	23	1.1e-02	44	6.4e-04	4
	TOPORS	5	2.0e-03	6	2.1e-03	8	3.4e-03	14	8.8e-07	3
	TCF20	7	2.6e-03	7	2.3e-03	17	8.4e-03	46	7.2e-04	6
	BCL9	9	3.8e-03	8	3.0e-03	16	7.6e-03	19	3.7e-05	3
	CHD7	8	3.2e-03	9	3.2e-03	11	4.6e-03	30	1.5e-04	7
	PDE4C	10	4.4e-03	10	3.3e-03	20	9.1e-03	25	7.9e-05	3
GBM	MED12L	2	7.7e-04	1	2.3e-05	8	1.1e-02	62	1.3e-02	3
	IDH1	1	2.9e-04	2	6.7e-05	2	4.8e-03	27	3.8e-03	15
	EMILIN1	3	1.7e-03	3	2.0e-04	45	3.2e-02	100	3.2e-02	4
	ATP6V0A4	4	1.9e-03	4	2.5e-04	77	4.7e-02	101	3.3e-02	4
	VARS2	5	4.8e-03	5	1.0e-03	64	4.1e-02	111	3.8e-02	3
	GALR1	6	5.5e-03	6	1.3e-03	119	7.0e-02	156	6.6e-02	3
	KCNH4	7	7.1e-03	7	2.0e-03	108	6.2e-02	125	4.6e-02	3
	CXorf22	8	7.1e-03	8	4.3e-03	47	3.3e-02	102	3.3e-02	8
	CYP4B1	13	1.0e-02	9	5.0e-03	168	1.0e-01	151	6.4e-02	3
	ITGAM	9	9.1e-03	10	5.3e-03	57	3.7e-02	109	3.7e-02	6
LUSC	ATXN10	2	3.9e-05	1	4.8e-05	117	3.2e-02	226	2.4e-02	3
	ZNF304	1	2.9e-05	2	5.6e-05	37	9.1e-03	164	1.1e-02	5
	C10orf79	4	5.7e-05	3	8.8e-05	16	4.8e-03	116	4.9e-03	9
	LINGO1	3	5.1e-05	4	1.1e-04	80	2.1e-02	208	1.9e-02	4
	OR5AS1	6	8.5e-05	5	1.4e-04	31	8.2e-03	144	8.6e-03	7
	SPANXN1	5	7.6e-05	6	1.5e-04	60	1.7e-02	188	1.6e-02	5
	SLC7A13	7	1.3e-04	7	2.2e-04	54	1.5e-02	201	1.8e-02	5
	CCDC85A	8	2.4e-04	8	3.1e-04	25	7.0e-03	141	8.0e-03	9
	DUSP27	10	3.9e-04	9	4.5e-04	14	3.9e-03	117	4.9e-03	11
	ZNF527	11	4.0e-04	10	5.0e-04	1	2.1e-04	12	6.0e-07	9
OV	DFNB31	1	4.1e-04	1	8.9e-05	5	1.7e-02	18	1.2e-02	4
	ERN2	2	5.9e-04	2	1.8e-04	13	2.6e-02	24	2.2e-02	5
	NCOA3	3	9.6e-04	3	3.0e-04	11	2.5e-02	21	1.8e-02	4
	CIC	4	2.5e-03	4	1.2e-03	18	3.1e-02	26	2.5e-02	4
	PKP4	5	3.4e-03	5	2.1e-03	20	3.4e-02	31	3.3e-02	5
	BRCA2	6	4.0e-03	6	3.0e-03	22	3.6e-02	32	3.5e-02	9
	OVGP1	11	1.1e-02	7	9.7e-03	38	6.0e-02	36	4.5e-02	4
	ADAR	9	1.1e-02	8	9.7e-03	68	9.6e-02	64	7.1e-02	4
	PCDH9	10	1.1e-02	9	9.9e-03	73	9.8e-02	66	7.4e-02	4
	MYST4	7	1.0e-02	10	1.0e-02	74	9.9e-02	71	8.0e-02	5
UCEC	CTGF	1	9.6e-05	1	8.3e-10	1	5.2e-04	6	3.7e-12	3
	KRT6C	2	7.0e-04	2	2.3e-05	2	1.0e-03	2	0<1e-16	3
	LSS	3	1.1e-03	3	2.8e-05	3	1.8e-03	3	7.6e-14	3
	EIF2C4	4	2.0e-03	4	3.7e-05	5	4.0e-03	8	9.6e-10	3
	CPNE8	5	2.4e-03	5	4.0e-05	6	4.8e-03	13	6.0e-09	3
	USP20	7	3.6e-03	6	5.3e-05	10	7.7e-03	19	3.6e-07	3
	RBM5	8	3.6e-03	7	5.3e-05	11	7.7e-03	20	3.7e-07	3
	SLC22A11	9	3.6e-03	8	5.4e-05	12	7.9e-03	21	4.2e-07	3
	PEG10	11	3.7e-03	9	5.5e-05	15	8.3e-03	24	5.8e-07	3
	HGF	10	3.7e-03	10	5.5e-05	14	8.3e-03	23	5.8e-07	3
