# Supplementary material for: Accurate Computation of Survival Statistics in Genome-Wide Studies
Source: PLoS Comput Biol. 2015 May 7;11(5):e1004071. doi: 10.1371/journal.pcbi.1004071 (PMC4423942; doi:10.1371/journal.pcbi.1004071)
Supplement: S4 Table — (PDF) [file pcbi.1004071.s005.pdf]

Table S4: The 10 genes with smallest p-values identified using the exact conditional test in cancer data. For each cancer type, we show the top 10 genes, their rank, and p-value using the exact permutational test, the asymptotic permutational test, the exact conditional test, and `R survdiff`. The number of samples with a mutation in the gene is also reported.

		exact permutational		asymp. permutational		exact conditional		R `survdiff`		
cancer type	gene	rank	p	rank	p	rank	p	rank	p	num. mut.
COADREAD	VPS13B	3	1.6e-03	9	2.5e-04	1	3.6e-04	1	6.2e-12	8
	ERBB4	4	1.6e-03	11	2.9e-04	2	5.9e-04	3	2.2e-10	8
	DOCK9	2	1.0e-03	2	4.6e-05	3	1.7e-03	5	4.3e-08	6
	PTPRM	1	5.5e-04	1	1.1e-05	4	2.3e-03	10	1.7e-05	8
	ZNF804B	7	4.5e-03	23	2.0e-03	5	2.9e-03	2	1.1e-10	5
	AQPEP	9	5.0e-03	24	2.3e-03	6	5.4e-03	4	2.5e-08	5
	CACNA1E	5	4.1e-03	12	4.4e-04	7	8.7e-03	21	1.4e-04	7
	DOCK3	6	4.2e-03	3	1.3e-04	8	1.1e-02	6	3.9e-06	3
	IGF1R	8	4.8e-03	4	1.5e-04	9	1.3e-02	7	8.9e-06	3
	ZNF568	10	5.4e-03	5	1.7e-04	10	1.5e-02	11	2.5e-05	3
KIRC	CDCA2	1	2.8e-04	1	2.8e-04	1	1.8e-04	7	6.2e-10	6
	FBXO43	25	1.1e-02	25	1.1e-02	2	2.5e-04	4	4.4e-16	5
	ATP10D	2	3.9e-04	3	1.2e-03	3	6.1e-04	5	3.6e-12	3
	EZH2	46	1.8e-02	54	2.5e-02	4	8.1e-04	2	<1.0e-16	3
	TDRD7	3	9.1e-04	4	1.5e-03	5	1.5e-03	10	5.0e-09	3
	PKD1L3	36	1.4e-02	36	1.6e-02	6	3.2e-03	13	6.5e-07	5
	RASEF	47	1.8e-02	64	2.9e-02	7	3.2e-03	6	5.5e-11	3
	TOPORS	5	2.0e-03	6	2.1e-03	8	3.4e-03	14	8.8e-07	3
	PCDH11X	49	1.8e-02	66	3.0e-02	9	4.2e-03	8	1.0e-09	3
	ZFAT	51	1.9e-02	68	3.0e-02	10	4.6e-03	9	2.6e-09	3
GBM	TBC1D2	32	3.0e-02	32	2.9e-02	1	3.7e-03	4	8.0e-05	5
	IDH1	1	2.9e-04	2	6.7e-05	2	4.8e-03	27	3.8e-03	15
	HDAC9	53	5.3e-02	57	6.2e-02	3	5.1e-03	1	6.1e-06	3
	ATP13A2	50	5.0e-02	50	5.5e-02	4	6.4e-03	8	3.7e-04	6
	CASKIN2	42	4.4e-02	42	4.7e-02	5	6.9e-03	5	1.6e-04	4
	GRM7	61	6.0e-02	64	6.9e-02	6	8.7e-03	2	7.3e-05	3
	MYST3	63	6.1e-02	65	6.9e-02	7	8.9e-03	3	7.9e-05	3
	MED12L	2	7.7e-04	1	2.3e-05	8	1.1e-02	62	1.3e-02	3
	BID	51	5.2e-02	51	5.5e-02	9	1.1e-02	12	7.1e-04	4
	NFYC	71	6.6e-02	73	7.4e-02	10	1.2e-02	6	2.0e-04	3
LUSC	ZNF527	11	4.0e-04	10	5.0e-04	1	2.1e-04	12	6.0e-07	9
	ZNF557	77	4.3e-03	94	7.7e-03	2	4.1e-04	3	2.0e-13	3
	CHTF18	895	1.1e-01	895	1.1e-01	3	5.2e-04	1	0<1e-16	4
	CNGA4	37	1.7e-03	33	2.2e-03	4	1.2e-03	14	2.3e-06	5
	BCL11A	304	2.9e-02	302	3.0e-02	5	1.3e-03	11	5.8e-07	7
	SIN3B	109	6.8e-03	126	9.7e-03	6	1.6e-03	6	1.0e-08	3
	ANKRD11	424	4.4e-02	427	4.5e-02	7	1.7e-03	7	2.4e-08	5
	RLTPR	628	6.9e-02	636	7.1e-02	8	1.9e-03	2	7.7e-14	3
	NARG2	115	7.7e-03	137	1.1e-02	9	2.2e-03	8	8.6e-08	3
	ATP2B2	149	1.1e-02	148	1.2e-02	10	2.4e-03	23	2.3e-05	7
OV	PABPC3	40	2.8e-02	39	2.9e-02	1	6.0e-03	2	1.0e-04	4
	KIAA0913	81	7.8e-02	81	8.5e-02	2	8.6e-03	1	6.2e-05	4
	NIPBL	115	1.3e-01	114	1.3e-01	3	1.2e-02	3	2.6e-04	5
	PCDHA9	46	3.6e-02	46	3.8e-02	4	1.2e-02	4	8.8e-04	4
	DFNB31	1	4.1e-04	1	8.9e-05	5	1.7e-02	18	1.2e-02	4
	RBM44	90	9.7e-02	91	1.0e-01	6	1.8e-02	5	1.0e-03	4
	TNRC18B	56	4.5e-02	55	4.6e-02	7	1.9e-02	7	3.0e-03	4
	PTCH1	66	5.7e-02	65	6.0e-02	8	2.1e-02	10	5.5e-03	6
	EFCAB6	125	1.4e-01	127	1.5e-01	9	2.1e-02	6	1.6e-03	5
	PCDH15	51	4.0e-02	50	4.2e-02	10	2.2e-02	12	6.6e-03	5
UCEC	CTGF	1	9.6e-05	1	8.3e-10	1	5.2e-04	6	3.7e-12	3
	KRT6C	2	7.0e-04	2	2.3e-05	2	1.0e-03	2	0<1e-16	3
	LSS	3	1.1e-03	3	2.8e-05	3	1.8e-03	3	7.6e-14	3
	TLR2	6	3.3e-03	43	1.2e-03	4	3.0e-03	7	3.7e-11	5
	EIF2C4	4	2.0e-03	4	3.7e-05	5	4.0e-03	8	9.6e-10	3
	CPNE8	5	2.4e-03	5	4.0e-05	6	4.8e-03	13	6.0e-09	3
	CFP	15	4.1e-03	46	1.5e-03	7	5.4e-03	14	2.8e-08	5
	TUT1	16	4.1e-03	47	1.5e-03	8	5.6e-03	15	4.1e-08	5
	SH3TC2	13	3.8e-03	29	4.4e-04	9	7.4e-03	18	2.3e-07	4
	USP20	7	3.6e-03	6	5.3e-05	10	7.7e-03	19	3.6e-07	3
