# Supplementary figures and images for: Accurate Computation of Survival Statistics in Genome-Wide Studies
Source: PLoS Comput Biol. 2015 May 7;11(5):e1004071. doi: 10.1371/journal.pcbi.1004071 (PMC4423942; doi:10.1371/journal.pcbi.1004071)

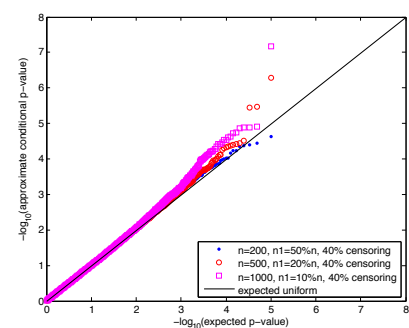

(a)

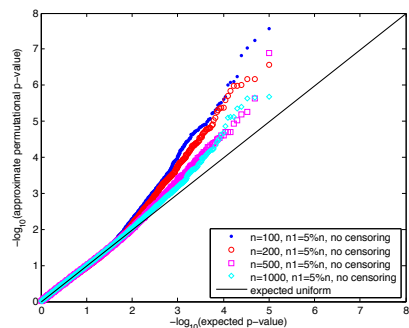

(b)

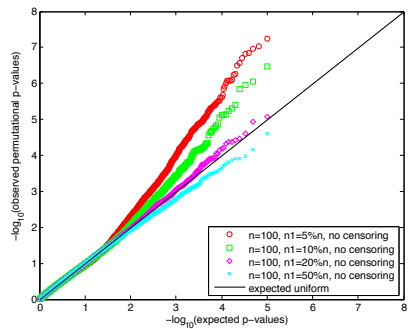

(c)

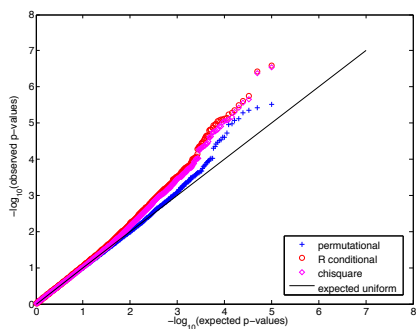

(d)

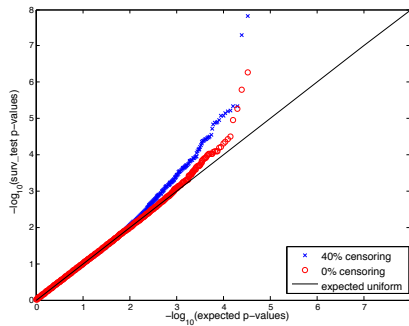

(e)

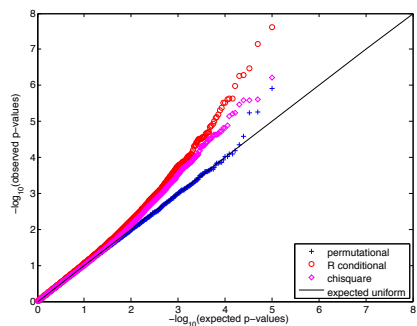

(f)

Supplement: S1 Fig — (a) Distribution of p-values obtained using the conditional approximation, and distribution of p-values for the uniform distribution. Generated considering 105 instances with n 1 = 100 samples in the small population, different number n of samples in total, and same survival distribution for all patients (≈ 40% censoring). (b) Distribution of p-values obtained using the permutational approximation, and distribution of p-values for the uniform distribution. Generated considering 105 data points with n 1 = 5%n samples in the small population, n total samples, and no censoring. (c) Distribution of p-values obtained using the permutational approximation, and distribution of p-values for the uniform distribution. Generated considering 105 data points with n = 100 total samples, different values of n 1, and no censoring. (d) Distribution of p-values obtained using different approximations, and distribution of p-values for the uniform distribution. Generated considering 105 instances with n = 500 total samples, n 1 = 5%n samples with a mutations in the gene, and same survival distribution for all patients (≈ 40% censoring). (e) Distribution of p-values obtained using surv_test method in coin R package, and distribution of p-values for the uniform distribution. Generated considering 105 instances with n = 500 total samples, n 1 = 5%n samples with a mutations in the gene, and same survival distribution for all patients, with 0% or 40% censoring. (f) Distribution of p-values obtained using different approximations, and distribution of p-values for the uniform distribution. Generated considering 105 instances with n = 500 total samples, n 1 = 5%n samples with a mutations in the gene, and same survival distribution for all patients ( ≈ 60% censoring). (PDF) [file pcbi.1004071.s007.pdf]

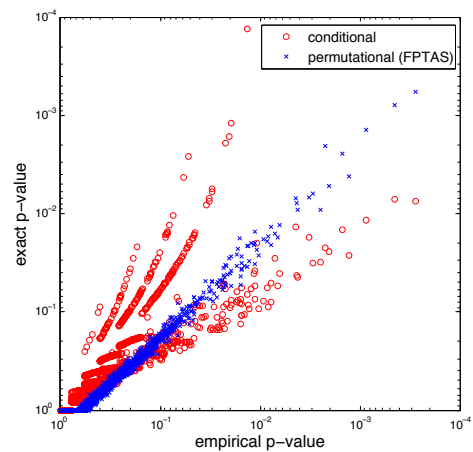

(a)

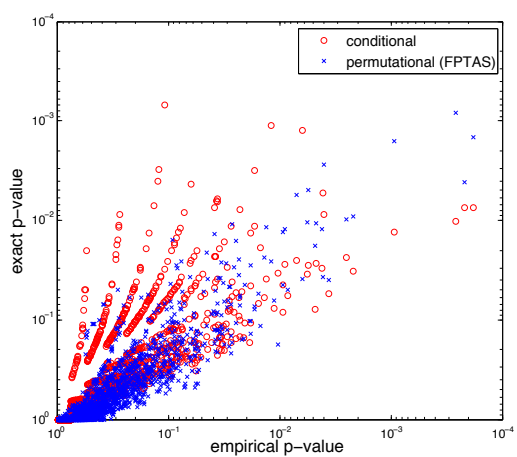

(b)

Supplement: S2 Fig — The R coefficients comparing the −log 10 exact p-values to the −log 10 empirical p-values are the following: in Fig. (a), permutational = 0.96, conditional = 0.88; in Fig (b), permutational = 0.72, conditional = 0.43. (a) Comparison of exact conditional p-values, exact permutational p-values, and empirical p-values for n = 100,n 1 = 5%n, and 30% censoring. Each point represents an instance of survival data. (b) Comparison of exact conditional p-values, exact permutational p-values, and empirical p-values for n = 100, expectation n 1 = 5%n, and 30% censoring. Each point represents an instance of survival data. (PDF) [file pcbi.1004071.s008.pdf]

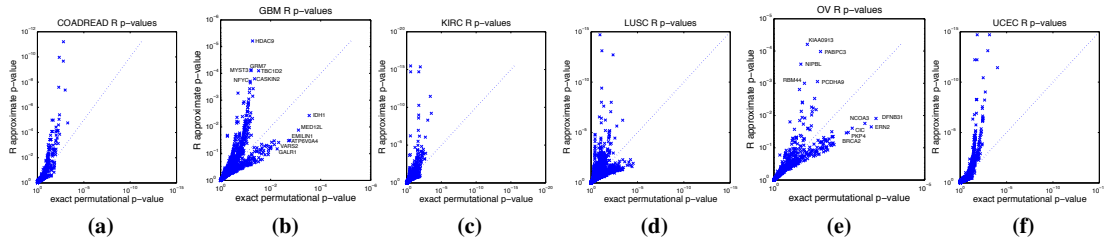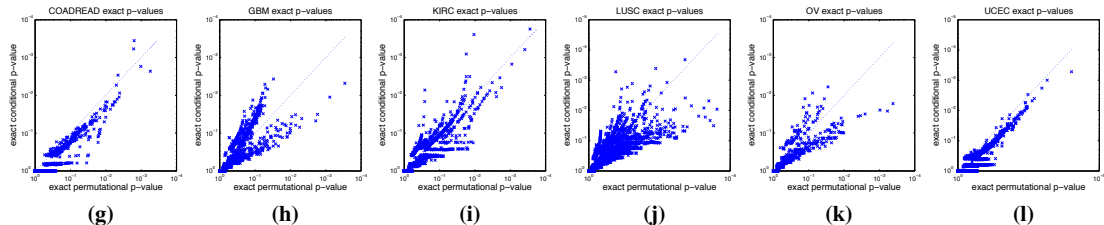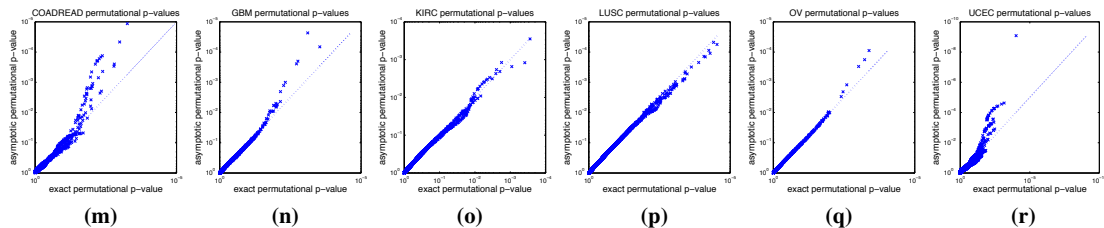

Supplement: S3 Fig — Comparison of the p-values from the exact permutational test, the exact conditional test, the asymptotic conditional approximation (as implemented in the survdiff function in R), and the asymptotic permutational approximation for cancer datasets COADREAD, GBM, KIRC, LUSC, OV, UCEC. (a,b,c,d,e,f): Each data point represents a gene, and the p-values computed using the exact permutational test and the p-values from R survdiff for the gene are shown. (g,h,i,j,k,l): Each data point represents a gene, and the p-values computed using the exact permutational test and the exact conditional test for the gene are shown. (m,n,o,p,q,r): Each data point represents a gene, and the p-values computed using the exact permutational test and the asymptotic permutational test for the gene are shown. (PDF) [file pcbi.1004071.s009.pdf]

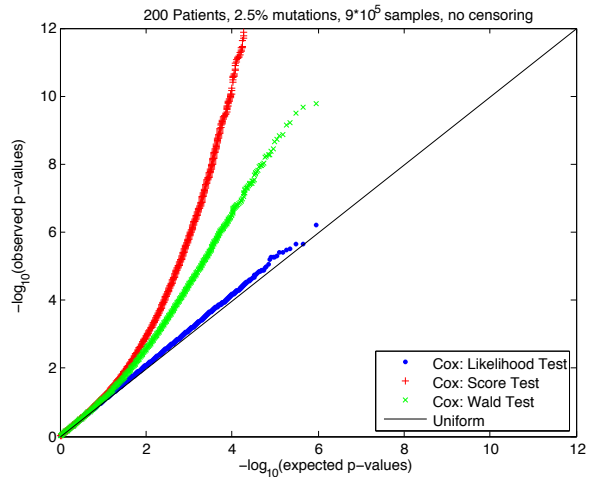

(a)

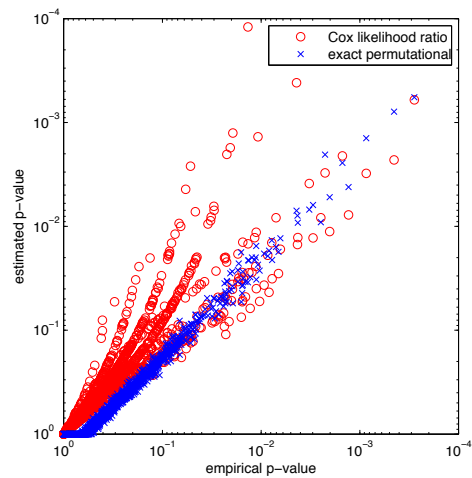

(b)

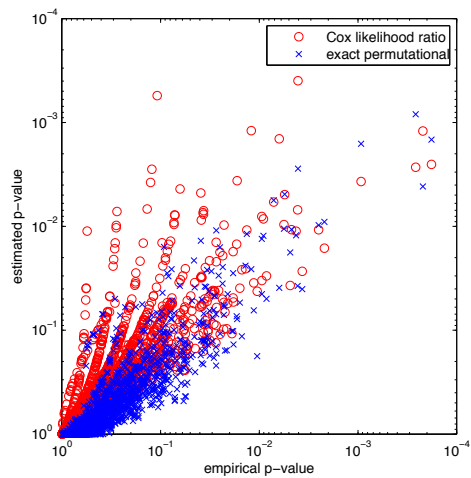

(c)

Supplement: S4 Fig — Comparison of the p-values from asymptotic approximations for the Cox Proportional-Hazard model and the uniform distribution, and comparison of the p-values from exact permutational tests and the Cox likelihood ratio test with the empirical p-values for two different null distributions. (a) Distribution of p-values obtained using the asymptotic approximation for the Cox Proportional-Hazard model and the distribution of p-values for the uniform distribution. Generated considering 9×10^5^ instances with n = 200 total samples, n 1 = 5 samples in the small population and same survival distribution for all patients (no censoring). (b) Comparison of Cox likelihood ratio p-values, exact permutational p-values, and empirical p-values for n = 100,n 1 = 5%n, and 30% censoring. Each point represents an instance of survival data. (c) Comparison of Cox likelihood ratio p-values, exact permutational p-values, and empirical p-values for n = 100, expectation(n 1) = 5%n, and 30% censoring. Each point represents an instance of mutations and survival data. The R coefficients comparing the −log 10 exact p-values to the −log 10 empirical p-values are the following: in Fig. (b), permutational = 0.96, Cox likelihood ratio = 0.70; in Fig (c), permutational = 0.72, Cox likelihood ratio = 0.46. (PDF) [file pcbi.1004071.s010.pdf]

End of iteration t

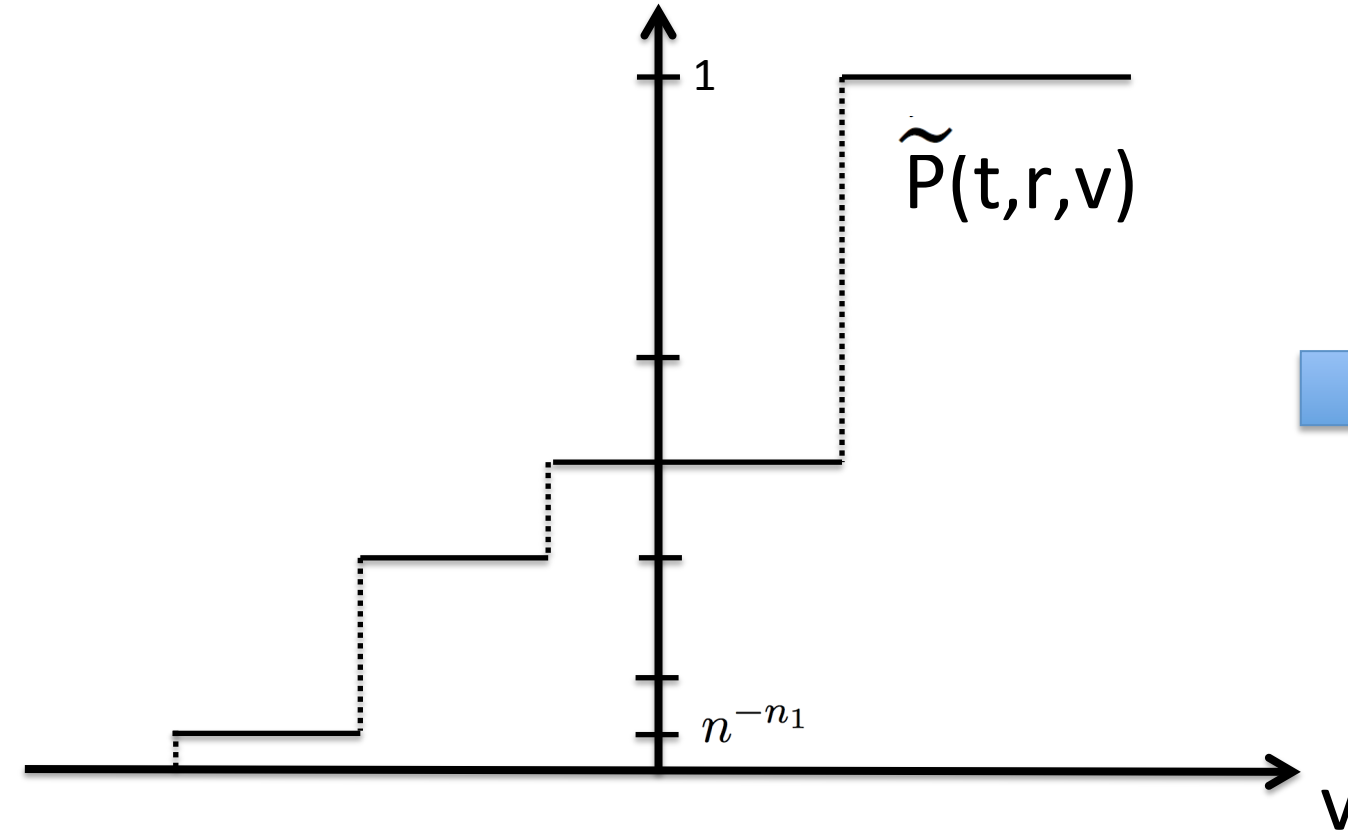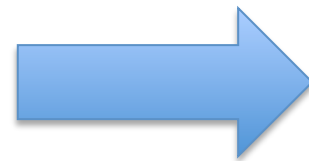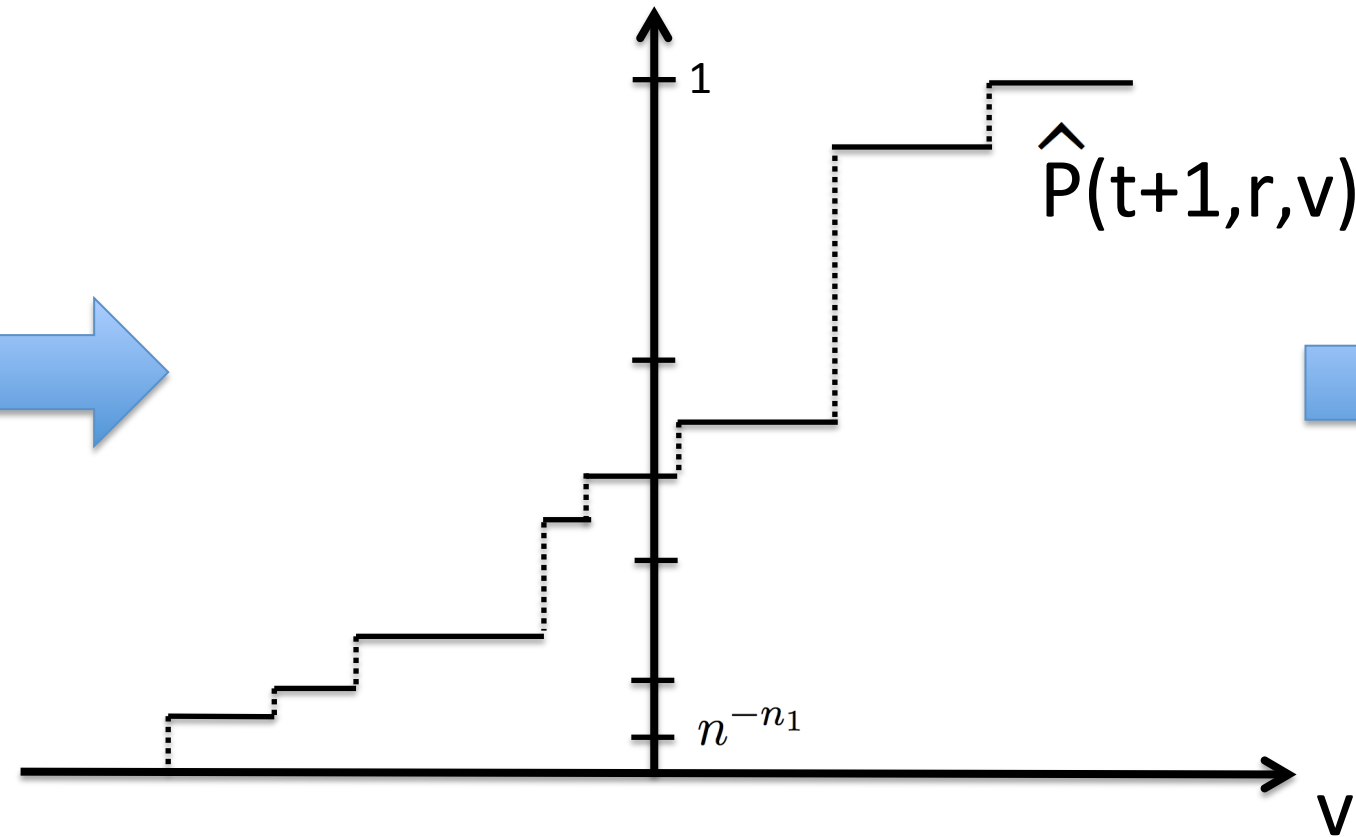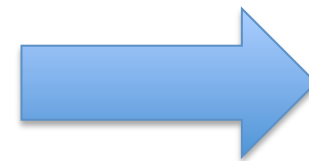

End of iteration t+1

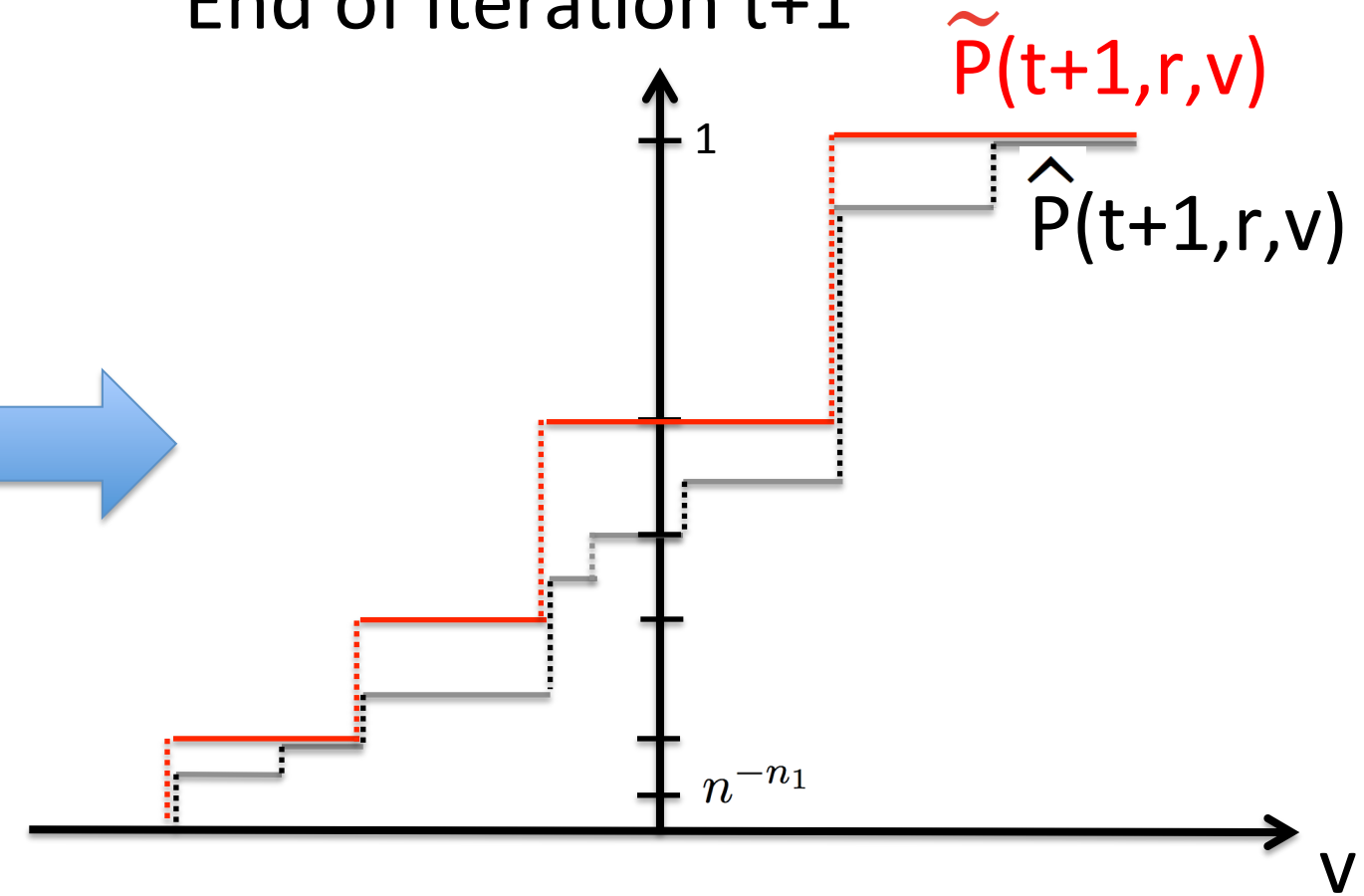

Supplement: S6 Fig — Starting from the approximation P˜(t,r,v) at time t that uses ℓ values of v to approximate P(t,r,v), in the t+1 iteration the FPTAS computes an approximation P^(t+1,r,v) for P(t+1,r,v) that uses up to 2ℓ values of v; then the approximation P˜(t+1,r,v) is built starting from P^(t+1,r,v) by appropriately reducing the number of values of v considered, while maintaining guarantees on the approximation. (PDF) [file pcbi.1004071.s012.pdf]

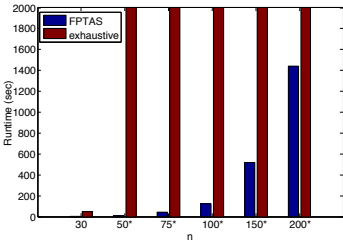

(a)

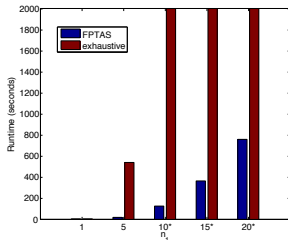

(b)

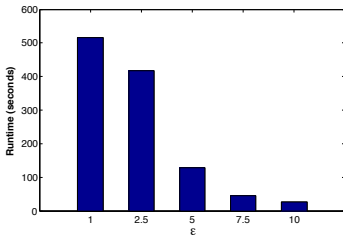

(c)

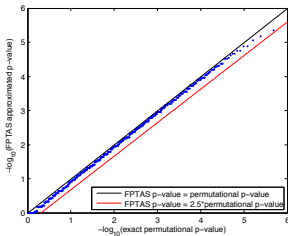

(d)

Supplement: S7 Fig — (a) Runtime of FPTAS and of the exhaustive enumeration for different values of n, and for n 1 = 10,ɛ = 5, no censoring. (b) Runtime of FPTAS and of the exhaustive enumeration for n = 100,ɛ = 5, no censoring, and different values of n 1. (c) Runtime of the FPTAS for different values of ɛ, and for n = 100,n 1 = 10, no censoring. (d) Comparison of the FPTAS p-values and the exact p-values (obtained with the complete enumeration algorithm) for n = 60,n 1 = 4, no censoring, and ɛ = 1.5. (PDF) [file pcbi.1004071.s013.pdf]
