# Supplementary material for: Accurate Computation of Survival Statistics in Genome-Wide Studies
Source: PLoS Comput Biol. 2015 May 7;11(5):e1004071. doi: 10.1371/journal.pcbi.1004071 (PMC4423942; doi:10.1371/journal.pcbi.1004071)
Supplement: S5 Fig — (Top) The log-rank test compares the Kaplan-Meier curves of the two groups. (Middle) Survival data is represented by sorting patients by increasing survival. x represents group labels for patients, and c represents censoring information (c i = 0 if event at time t i is censored, c i = 1 otherwise). (Bottom) The conditional test is defined by a series of independent contingency tables with marginals corresponding to the number of patients at risk in each group and the number of events in each group, conditioning on the patients at risk at each non-censored time; O i denotes the number of events at time t i, R i,j denotes the number of patients at risk in group j at time i. The permutational test considers all (nn1) possible locations of the n 1 patients with label 1 in the vector x. (PDF) [file pcbi.1004071.s011.pdf]

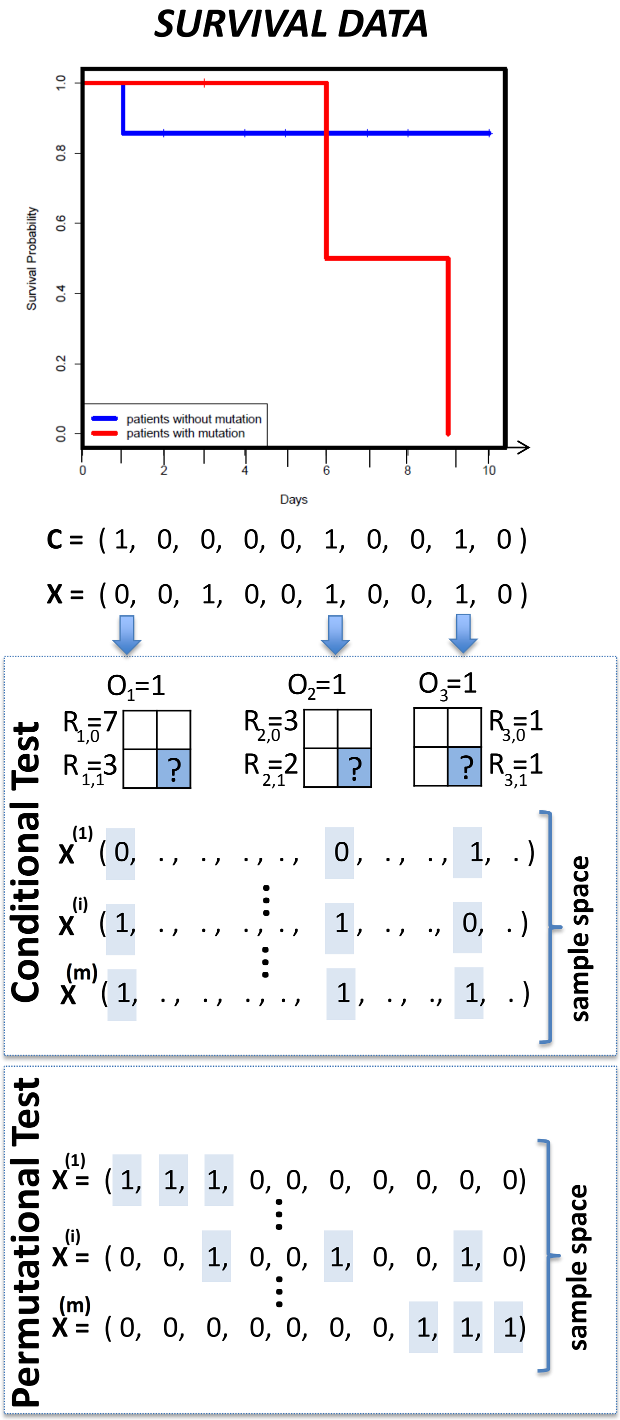
SURVIVAL DATA
Survival Probability
1.0
0.8
0.6
0.4
0.2
0.0
0
2
4
6
8
10
Days
patients without mutation
patients with mutation
C = (1, 0, 0, 0, 0, 1, 0, 0, 1, 0)
X = (0, 0, 1, 0, 0, 1, 0, 0, 1, 0)
Conditional Test
O1=1
R1,0=7
R1,1=3
O2=1
R2,0=3
R2,1=2
O3=1
R3,0=1
R3,1=1
?
X(1) (0, . , . , . , . , 0 , . , . , 1, .)
X(i) (1, . , . , . , . , 1 , . , ., 0, .)
X(m) (1, . , . , . , . , 1 , . , ., 1, .)
sample space
Permutational Test
X(1) = (1, 1, 1, 0, 0, 0, 0, 0, 0, 0)
X(i) = (0, 0, 1, 0, 0, 1, 0, 0, 1, 0)
X(m) = (0, 0, 0, 0, 0, 0, 0, 1, 1, 1)
sample space
